# Molecular cloning, in-silico characterization and functional validation of monodehydroascorbate reductase gene in *Eleusine coracana*

**DOI:** 10.1371/journal.pone.0187793

**Published:** 2017-11-27

**Authors:** Bhawana Negi, Prafull Salvi, Deepesh Bhatt, Manoj Majee, Sandeep Arora

**Affiliations:** 1 Department of Molecular Biology and Genetic Engineering, College of Basic Sciences and Humanities, G. B. Pant University of Agriculture and Technology, Pantnagar, Uttarakhand, India; 2 National Institute of Plant Genome Research, Aruna Asaf Ali Marg, New Delhi, India; National Botanical Research Institute CSIR, INDIA

## Abstract

Ascorbic acid is a ubiquitous water soluble antioxidant that plays a critical role in plant growth and environmental stress tolerance. It acts as a free radical scavenger as well as a source of reducing power for several cellular processes. Because of its pivotal role in regulating plant growth under optimal as well as sub-optimal conditions, it becomes obligatory for plants to maintain a pool of reduced ascorbic acid. Several cellular processes help in maintaining the reduced ascorbic acid pool, by regulating its synthesis and regeneration processes. Current study demonstrates that monodehydroascorbate reductase is an important enzyme responsible for maintaining the reduced ascorbate pool, by optimizing the recycling of oxidized ascorbate. Cloning and functional characterization of this important stress inducible gene is of great significance for its imperative use in plant stress management. Therefore, we have cloned and functionally validated the role of monodehydroascorbate reductase gene (*mdar*) from a drought tolerant variety of *Eleusine coracana*. The cloned *Ecmdar* gene comprises of 1437bp CDS, encoding a 478 amino acid long polypeptide. The active site analysis showed presence of conserved Tyr_348_ residue, facilitating the catalytic activity in electron transfer mechanism. qPCR expression profiling of *Ecmdar* under stress indicated that it is an early responsive gene. The analysis of *Ecmdar* overexpressing *Arabidopsis* transgenic lines suggests that monodehydroascorbate reductase acts as a key stress regulator by modulating the activity of antioxidant enzymes to strengthen the ROS scavenging ability and maintains ROS homeostasis. Thus, it is evident that *Ecmdar* is an important gene for cellular homeostasis and its over-expression could be successfully used to strengthen stress tolerance in crop plants.

## Introduction

Ascorbic acid is a vital antioxidant molecule that plays a crucial role in removal of excessive reactive oxygen species (ROS), by enzymatic as well as non-enzymatic scavenging mechanisms [1]. It participates in numerous developmental processes such as cell division, cell expansion and cell wall growth [2–3]. It is widely reported that ascorbate is used as a crucial cellular factor for inducing tolerance against abiotic stresses such as drought, temperature, salinity, ozone and high light intensity [4–9]. A high redox status of ascorbate in plant cells is required for adaptation of plants to environmental stresses [1]. Reduced ascorbate pool is maintained in cells through synthesis, recycling and transportation [10]. Ascorbate is oxidized to monodehydroascorbate (MDHA) and then to dehydroascorbate (DHA), by enzymatic action of ascorbate peroxidase (APX) and ascorbate oxidase (AAO) respectively and non-enzymatic reaction like detoxification of toco-trienoxyl radical during the process of ROS scavenging [11]. Oxidized forms of ascorbate i.e. MDHA and DHA are converted back to reduced ascorbate by monodehydroascorbate reductase (MDAR) using NADH or NADPH as electron donor and by dehydroascorbate reductase (DHAR) using reduced glutathione (GSH) as an electron donor, respectively [12]. Monodehydroascorbate, a radical, is the primary product of AsA oxidation in ROS scavenging reactions, which is further oxidized to dehydroascorbate by non-enzymatic reaction. Further, DHAR utilizes two molecules of NADPH+ H^+^ in the process of reduction of DHA to ascorbate, whereas MDAR utilizing only one molecule of NADPH. Therefore, regeneration of reduced ascorbate via MDAR is an economical option for the stressed plants. Thus, monodehydroascorbate reductase (MDAR EC 1.6.5.4) appears to be an important enzyme regulating the reduced AsA pool [13], and is a key component in the stress tolerance profile of a plant.

Finger millet (*Eleusine coracana*) is a stress-hardy crop, having wide adaptability for environmental conditions, as it grows in different climatic regions of Africa and Asia. In India, it is cultivated in different climatic zones such as Karnataka, Andhra Pradesh (near to sea level) and in hills of Uttarakhand (1600 m above sea level) [14,15]. Finger millet possesses highly active antioxidant machinery, making this crop capable of withstanding unfavorable conditions. Although varietal differences, towards the stress tolerance potential, are known to exist in finger millet, specific abiotic stress tolerant varieties can be used for allele mining of important genes [16]. Limited information is available in finger millet, towards cloning and stress tolerance profiling of *mdar* through over-expression studies [17–19]. Further, partial *Ecmdar* sequence information available in database (UniP ID-D3K3M7, NCBI–HQ625516), provides only a diminutive view about the functional and structural annotation of monodehydroascorbate reductase. Therefore, in the present study, attempts were made to clone the monodehydroascorbate reductase gene (*mdar)*, from a drought tolerant variety of finger millet (PR202), and perform its structural annotation using *in*-*silico* tools and functional validation through overexpression in *Arabidopsis*. Further, real time quantitative PCR studies were carried out to validate the temporal expression of *Ecmdar* under water deficit, UV-C and salinity stress conditions.

## Material and methods

### Plant material

Seeds of finger millet variety PR202, a drought tolerant genotype, were obtained from Ranichauri campus of G B Pant University of Agriculture and Technology. Surface sterilized seeds were sown in pots and transferred to plant growth chamber under controlled conditions (light/dark regime of18/6 h at 25°C, and light intensity of 3040 lux) and allowed to grow for 15 days.

### Stress treatment

Seeds of *E*. *coracana* (variety PR-202) were washed with Tween-20 detergent for 5 minutes and then thoroughly washed under running tap water for 20 minutes. The seeds were then surface sterilized with 0.1% HgCl_2_ and washed with autoclaved distilled water.

Surface sterilized seeds were germinated in germination bottles in presence of half strength MS media, without stress. 10 seeds per bottle were placed in each germination bottle on germination paper and kept in dark for 48 hours at 30°C. Germinated seeds were then grown for fifteen days by transferring them to plant growth chamber under controlled conditions (light/dark regime of 18/6 h at 25°C, and light intensity of 3040 lux) and allowed to grow for 15 days. Half of the fifteen day old seedlings were then subjected to water deficit stress by transferring them to germination bottles containing PEG-6000 at -1.5MPa. The seedlings were exposed to water deficit stress for different time intervals and samples were collected after 0.5, 1, 2, 6, 12, 48, 96 hours.

For ultra-violet radiation treatment, seedlings were grown on MS media in culture bottles and ten surface sterilized seeds were inoculated in each culture bottle. Cultures were maintained under sterile conditions (light/dark regime of 18/6 h at 25°C, and light intensity of 3040 lux) in tissue culture laboratory. For providing UV stress, 15-day-old seedlings were exposed to UV-C radiations (252nm, 50 kJ/m^2^), provided by 30W germicide fluorescent lamp (Lab Companion, BC-11, Korea) for increasing intervals of time. Samples were taken at different time intervals 30, 60, 120 and 240 min and analysed for gene expression.

In order to check the effect of salinity stress on expression of *mdar* gene, surface sterilized seeds were allowed to germinate and grow on MS media supplemented without (control) and with 50, 100 and 200mM NaCl, in germination bottles. Ten seeds were inoculated in each bottle, and incubated in tissue culture room under controlled conditions (light/dark regime of 18/6 h at 25°C, and light intensity of 3040 lux) for 15 days. 15-day-old seedlings were used for qPCR studies.

#### Real time PCR analysis of *mdar* transcript

Total RNA was isolated from seedlings by RNA express reagent (Hi Media (P) Ltd.) as per manufacturer’s protocol. Total RNA (1000μl) was used to synthesis c-DNA by Verso cDNA synthesis Kit (Thermo Scientific India Pvt. Ltd., Mumbai). To perform qPCR, 12 μl of PCR reaction mixture was prepared, containing 2X SYBR^®^ Premix Ex Taq^TM^buffer (Takara Clontech, TAKARA BIO INC Shiga Japan), 0.3μl of primers (0.25pmol/μl of each primer), 1μl of cDNA and molecular grade water. Amplification conditions of *Ecmdar* under qPCR were as follows: initial denaturation at 95°C for 3min, followed by 40 cycles of denaturation at 95°C for 45 sec, 59°C for 30 sec, 72°C for 35 sec. qPCR was performed by ABI Step One real-time thermocycler. All samples were tested in triplicates, for endogenous gene as well as the gene of interest. β-actin was used as endogenous control.

### Isolation and molecular cloning of the *mdar* gene from *E*. *coracana*

Total RNA from finger millet seedlings was isolated by using RNA X-press reagent (Hi-media) according to the manufacturer’s protocol and quantified by Nano-drop spectrophotometer. Total RNA was used to synthesize single strand cDNA using oligodT primers (Thermo Fisher Scientific India Pvt. Ltd., Mumbai). Monodehydroascorbate reductase gene specific primers were designed from the conserved sequences of *mdar* gene obtained by multiple sequence alignment of *mdar* genes from related monocot species, using online tool clustal omega (http://www.ebi.ac.uk).

Partial *Ecmdar* gene fragment (500bp) was cloned into pGEM-T Easy vector (Promega, Madison WI, USA) and sequenced. Rapid amplification of cDNA ends (RACE) was done for full length isolation of *mdar* gene. For 5’ RACE, four reverse primers were designed from partial *mdar* sequence and kit specific anchor primer was used as forward primer (Table 1). For 3’RACE, three forward primers were designed from partial mdar sequence and kit specific primer used as reverse primer (Table 1). Finally, 3’and 5’RACE was performed using 5’, 3’RACE kit (Second generation, Roche). PCR products of 3’ and 5’ RACE were cloned in pJET 1.2 cloning vector and sequenced. Sequences from partial, 3’RACE and 5’RACE products were aligned by SeqManII DNASTAR offline tool and a full length cDNA sequence was generated. Further, a pair of primers mdarorf forward and mdarorf reverse (Table 1) were designed from these overlapping sequences, so as to obtain the full-length CDS using cDNA as a template. Full length CDS sequence of *Ecmdar* gene was submitted to the GenBank database (Accession number KT230521.1).

**Table 1:** List of primers used.

Name	Sequence (5’– 3’)	Purpose
MDAR1 Forward	CTTGCGTTGGTGCTAATGATGAGT	*mdar* partial gene amplification
MDAR1 Reverse	CAACGTGAAGACCCTGGAGTAGAA	*mdar* partial gene amplification
RACE Forward1	AGCCTTGGTTACTAACAAGATAAGG	3’ RACE for *mdar*
RACE Forward2	AGGTCACAGCAGTAATCCTGAAAGA	3’ RACE for *mdar*
RACE Forward3	GTAAATGGACAAATGCAGACAAGCG	3’ RACE for *mdar*
RACE Reverse1	CAGTTCTACGAGCAGTCAACATG	5’ RACE for *mdar*
RACE Reverse2	CTAGTATTTGCACGGATACCGATAC	5’ RACE for *mdar*
RACE Reverse3	ATACACAGAGCTTTCGCTTGTCTGC	5’ RACE for *mdar*
RACE Reverse4	AGACCATGGTGACCCTTATCTTGTTA	5’ RACE for *mdar*
PCR ANCHOR	GACCACGCGTATCGATGTCGAC	5’& 3’ RACE for *mdar*
OligodT Anchor	GACCACGCGTATCGATGTCGACTTTTTTTTTTTTTTTTV	5’& 3’ RACE for *mdar*
*mdarorf* Forward	GAGCAACCGACGGAA	Full length *mdar* CDS
*mdarorf* Reverse	CAAACCTTCACCACCTG	Full length *mdar* CDS
*mdar* full length with BamHI sites Forward	GGATCCAATGGGGCGCGCGTTCGTGTACGTG	To clone *mdar* in bacterial expression vector
*mdar* full length with EcoRI sites Reverse	GAATCCACCTGCGGCGCTTCCTGCCATA	To clone *mdar* in bacterial expression vector
RT Forward Primer	CTTGTGTTGGTGCTAATGATGAGT	Real-time PCR for *mdar*
RT Reverse Primer	AGCTGTAACCAATGCCTTGC	Real-time PCR for *mdar*
Actin Forward	ATTCTTACCCTCAAGTATCC	For endogenous gene regulator
Actin Reverse	CATGATCTGAGTCATCTTCT	For endogenous gene regulator

### *In-silico* analysis of cloned *Ecmdar*

The *Ecmdar* nucleotide sequence was subjected to homology search using Basic Local Alignment Search Tool of NCBI (http://www.ncbi.nlm.nih.gov). The sequence was translated into six possible reading frames using online translation tool (web.expasy.org/translate/). The MDAR protein sequence was also submitted to pBLAST to search for homology with other MDAR amino acid sequences available in the NCBI database. Conserved domain analysis of MDAR amino acid sequence was done to find out the functionally conserved domain(s) via NCBI CCD tool file://localhost/(https/::www.ncbi.nlm.nih.gov:Structure:cdd:wrpsb.cgi). A phylogenetic tree was constructed by Molecular Evolutionary Genetic Analysis (MEGA) software, version 4.0 using neighbor joining method with complete deletion and Poisson correction settings. Expasy-protpraram server was used for physiochemical characterization of *Ec*MDAR protein. Molecular weight, theoretical isoelectric point (pI), total number of positive and negative residues, extinction coefficient, instability index, aliphatic index (AI) and grand average hydropathy (GRAVY) parameters were analyzed (http://web.expasy.org/cgi-bin/protparam/export). Trans-membrane helix prediction of *Ec*MDAR was done through TMHMM server 2.0 (www.cbs.dtu.dk). Potential post translational modification sites were analyzed through NetPhos2.0 and NetNGly 1.0 server. Structural and functional annotation of *Eleusine coracana* MDAR protein sequence was done by Predicted Protein online tool (http://www.predictprotein.org). The functional and structural annotation of the protein provides predicted information about secondary structure, solvent accessibilities, amino acid composition and presence of trans-membrane helix. Prediction potential disulfide bonding site was done by DiANNA 1.1 online tool (http://clavius.bc.edu/~clotelab/DiANNA/). 3D model of *Ec*MDAR protein was computed by Swiss model web server (https://swissmodel.expasy.org/), through homology template approach. Validation of the predicted 3D model of *Ec*MDAR was done by Errate and Verify 3D. Ramachandran plot analysis of the *Ec*MDAR 3D model was done by RAMEPAGE web server (http://mordred.bioc.cam.ac.uk/~rapper/rampage). Active site and ligand binding site prediction of *Ec*MDAR was done by 3DLigandsite (www.sbg.bio.ic.ac.uk/3dligandsite). A gene to gene interaction analysis for Ec*mdar* was done through string based analysis (http://string-db.org). The promoter prediction tool 2.0 (http://www.cbs.dtu.dk/services/promoter) was used to probe the 5’upstream region of the *mdar* gene for potential promoter analysis. Subsequently, to find out stress responsive cis regulatory elements in the *mdar* promoter region, a putative ~1 kb promoter region of *Oryza sativa mdar* was analyzed by PLANTCARE online server tool (bioinformatics.psb.ugent.be/webtools/plantcare/html/).

### Bacterial expression of *Ecmdar*

*Ecmdar* CDS was amplified by a set primers containing EcoRI and BamHI restriction sites. The amplified vector was clone into pET23b vector (Novagen, Madison, WI, USA) and transformed into *E*.*coli* DH5α cells. The positive clones were confirmed by colony PCR, restriction digestion, and followed by sequencing. Plasmid was isolated from positive clone of *E*. *coli* DH5α and transformed into *E*. *coli* BL21 (DE3) expression host cells. The transformed *E*.*coli* BL21(DE3) expression host cells were grown in 5ml of LB media supplemented with 0.3mM IPTG at 22°C for 16hrs at 120 rpm. After that, cells were pellet down by centrifugation and washed with 20mM sodium phosphate buffer (pH 7.6) containing 10mM β-Mercaptoethanol. Subsequently, the cells were re-suspended in lysis buffer (20mM Tris HCl buffer (pH-7.6), 10mM β-ME, 0.5mM NaCl and 20μl protease inhibitor) and lysed by sonication. Sonication was done with 3 pulses of 20sec each at 70% power with a gap of 25sec between each pulse and then centrifuged at 13,000g for 10 minutes at 4°C. The soluble and insoluble fractions were analyzed on 12% SDS-PAGE.

### Functional validation of *Ecmdar*

For functional validation of *Ecmdar* in plants, an over-expression construct of *Ecmdar* was transferred into *Arabidopsis thaliana*. *Agrobacterium* mediated in-planta transformation method was used for transformation. Performance of transgenic lines was compared under control and 100mM NaCl induced salinity stress conditions, for functional validation of *Ecmdar*.

#### Vector construction for plant transformation

Total RNA was extracted from *Eleusine coracana* leaves with RNA-Xpress reagent (Hi-Media, India) according to the manufacturer’s instructions. Total RNA (1 μg) was reverse-transcribed into cDNA using the Verso two step cDNA synthesis kit (Thermo Fisher Scientific, USA). Full-length cDNA of *Ecmdar* (GenBank accession number: KT230521) was obtained by gene specific primers containing *attB* recombination site (*mdar* plant vector Forward: CACCATGGGGCGCGCGTTCGTGTACGTG and *mdar* plant vector Reverse: AAACCACCTGCGGCGCTTCCTGCCATA), and then this amplicon was ligated in to pEarleyGate 103, under the control of CaMV 35S promoter, by using Invitrogen gateway technology (S1 Fig). The recombinant plasmid 35S::*Ecmdar* was introduced into the *Agrobacterium tumefaciens* strain GV3101. The *Agrobacterium* mediated floral dip in-planta transformation method was used to transfer the construct into *Arabidopsis thaliana* (Col 0). Independent transgenic lines were selected through BASTA resistance.

#### Quantitative expression analysis of *Ecmdar*

Expression analysis of *Ecmdar* gene in T_1_ generation transgenic plants was done by qPCR. Total RNA was isolated from the leaves of transgenic and wild type plants at vegetative stage. Real time PCR (qPCR) was performed in Step-one-plus PCR (Applied Bioscience), using SYBR green chemistry.

#### Stress treatment to *Arabidopsis* plants

Surface sterilized seeds from transformed and wild type *Arabidopsis* plants were grown in pots containing soil:vermi compost mixed in a ratio of 2:1(v/v). At vegetative stage, one set of seedlings were subjected to salt stress by irrigating them with 100ml of 100mM NaCl solution, on alternative days, while the control seedlings were irrigated with normal water. Samples were collected on 6^th^ day. Standard biochemical stress markers were analyzed, to evaluate the performance of transgenic plants under stress.

Various biochemical stress markers were analyzed to study the effect of over-expression of *Ecmdar* gene in transgenic and wild type plants.

#### Free proline content

Free proline was determined according to [20]. Leaf sample (1 g) was homogenized in 10mL of 3% sulfosalicylic acid and the homogenate was centrifuged at 10000g for 30 minutes at room temperature. 1mL of supernatant was mixed with 1mL of glacial acetic acid and 1mL of acid ninhydrin reagent and the reaction mixture were incubated at 100°C for 1h. The reaction was terminated in an ice bath. The chromophore from the reaction mixture was extracted in 2mL of toluene and its absorbance measured at 520nm. Concentration of proline in the sample was calculated from a standard curve of L-proline.

#### Malondialdehyde content

The procedure given by [21] was followed for measuring the malondialdehyde (MDA) content. Leaf sample (1 g) was homogenized in 10mL of 0.25% TBA (w/v) prepared in 10% TCA. The homogenate was then incubated at 95°C for 30min. The resultant mixture was centrifuged at 10000g for 30min. Absorbance of the supernatant was measured at 532 and 600nm. Absorbance at 600nm was subtracted from the absorbance at 532nm, for non-specific interference. The concentration of MDA was calculated by using an extinction coefficient 155 mM^−1^cm^−1^.

#### Electrolyte leakage

Electrolyte leakage (EL) was estimated according to the method of [22]. Fresh leaf samples (1 gm) were washed with triple distilled water and cut into small pieces (5 mm^2^ segments) and suspended in test tubes containing 15ml of de-ionized water for 25 minutes. Tubes were incubated in a water bath at 25°C for 2h. After incubation, electrical conductivity (EC_1_) of the bathing solution was recorded with an electrical conductivity meter (Eutech Instruments, Singapore). These samples were then kept at 100°C for 30 minutes to completely disintegrate the tissues and release the electrolytes. Samples were then cooled, and final electrical conductivity (EC_2_) was measured. The per cent leakage of electrolytes was calculated using the formula: *Electrolyte Leakage* = 1 − (*EC*1 ÷ *EC*2) × 100.

#### Chlorophyll content

Chlorophyll content of control and treated plants was determined by using the method of [23]. For chlorophyll extraction, 75mg of fresh leaf discs of uniform diameter were immersed in 10ml of dimethylsulphoxide (DMSO). Tubes were incubated at 65°C for 4 hrs in a water bath. After incubation, the absorbance of the resultant solution was measured at 663 and 645nm, with DMSO as blank. The concentration of Chl.a, Chl.b and total Chlorophyll (mg g^-1^ FW) was determined using the following equations:

Chla=[(12.7×A663)−(2.63×A645)]×extractvolume÷wt.ingram×1000

Chlb=[(22.9×A645)−(4.68×A663)]×extractvolume÷wt.ingram×1000

TotalChlorophyll=[(20.2×A645)+(8.02×A663)]×extractvolume÷wt.ingram×1000

#### Chlorophyll fluorescence (F_V_/F_m_)

Chlorophyll fluorescence in transgenic and wild type plant leaves was measured at room temperature (26°C) and ambient CO_2_ concentration. Measurements were made using an actinic light of 3,000 μmol photons m^−2^s^−1^, 4s flashes, after dark adapting the leaves for 30min. The fluorescence parameters evaluated were: F_0_, initial fluorescence, Fm, maximal fluorescence and the Fv/Fm ratio, representing the quantum efficiency of PS-II, where Fv is the variable fluorescence (Fm–F_0_).

#### Antioxidant enzyme activity

Leaf samples (0.5g fresh weight) were homogenized in a pre-chilled pestle mortar in 6ml ice-cold extraction buffer containing 50mM KH_2_PO_4_–KOH (pH 7.5), 0.1mM ethylene diamine tetraacetate, 0.3% (v/v) Triton X-100 and 2% (w/v) insoluble polyvinyl polypyrrolidone-40. The homogenate was kept on ice for 10min, and then centrifuged at 13,000g for 15min at 4°C. An aliquot of supernatant was used for determination of enzyme activity.

APX: Ascorbate peroxidase activity was determined according to [24]. For enzyme assay, 60μl of supernatant was mixed with 1,438μl of assay buffer [50mM phosphate buffer (pH 6.0), 0.1μM EDTA, 0.5mM ascorbate] and 2μl of 0.5M H_2_O_2_ was added to start the reaction. The decrease in absorbance was recorded and enzyme activity was calculated by using extinction coefficient 2.8mM^−1^cm^−1^ at 290nm. Specific enzyme activity was expressed as enzyme units per milligram of protein.

MDAR: Specific activity of Monodehydroascorbate reductase was assayed as described by [25]. The assay was performed at 340nm (using absorbance coefficient of 6.2mM^−1^cm^−1^) in a 2ml reaction mixture containing 50mmol L^−1^ Potassium phosphate buffer (pH 7.0), 0.2mmol L^−1^NADH, 2.5mmol L^−1^ ascorbate, 0.25U ascorbate oxidase and 60μl of enzyme extract. The decrease in absorbance due to ascorbate oxidase-induced oxidation of NADH was followed at 340nm. Specific enzyme activity in various samples was calculated in terms of μmol of NADH oxidized per minute, and expressed as enzyme units per mg of protein.

DHAR: The enzyme activity was measured as described by [26], with the reaction mixture comprising of 50mM morpholinoethanesulfonate buffer (pH 6.3), 2mM dehydroascorbate, 5mM GSH and enzyme extract. The increase in absorbance at 290nm was measured and the activity calculated. Specific enzyme activity was expressed as enzyme units per milligram of protein.

### Statistical analysis

The data presented are mean values ± SE. Measurements were performed on three replicates for each treatment (n = 3). The data was submitted to factorial analysis of variance and Duncan test was used to compare the differences between means using least significant differences at P ≤ 0.05.

## Results and discussion

For optimum plant growth a dynamic equilibrium between the reduced and oxidized forms of ascorbate is essential, as it regulates cellular processes such as cell growth, cell wall expansion, cell division and scavenging of ROS. Monodehydroascorbate reductase, an antioxidant enzyme, plays a crucial role in maintaining the reduced form of ascorbate in cells. There are various isoforms of Monodehydroascorbate reductase that exist in a cell; out of which cytosolic and peroxisomal isoforms are considered to be the regulatory points for maintaining ascorbate pool [27]. In our endeavor to understand the dynamics of this essential process, we have cloned and characterized the *mdar* from a stress tolerant variety of *Eleusine coracana*.

### Isolation and molecular cloning of *mdar* gene from *E*. *coracana*

**Fig 1:**
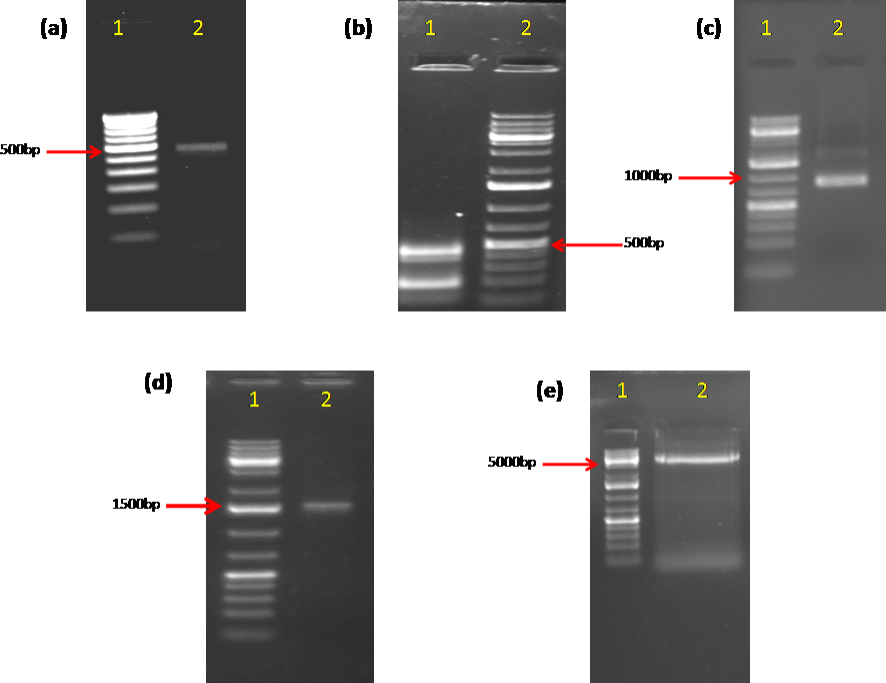
Isolation and molecular cloning of *Ecmdar*cDNA. a) PCR amplification using conserved *mdar* primers: amplification of a 500bp amplicon from conserved *mdar* primers L1–100 bp DNA ladder; L2–460 bp PCR amplicon. b) 5’ RACE: L1–400 bp amplicon of 5’RACE; L2–1 kb-plus DNA ladder c) 3’RACE: L1–1 kb-plus DNA ladder; L2–816 bp amplicon of 3’RACE PCR amplicon. d) Full length clone of *Ecmdar*: L1–1 kb-plus DNA ladder; L2—~1500 bp full length amplicon of *Ecmdar*. e) Full length genomic sequence of *Ecmdar*: L1–1 kb-plus DNA ladder; L2—~5000 bp full length amplicon of genomic *Ecmdar*.

A partial fragment of *mdar* was amplified by gene specific primers, designed from the conserved regions of *mdar* gene of related monocot families. A partial sequence of 500bp was amplified by using cDNA as a template and sequenced. The sequence information of partial fragment was utilized in designing of 3’and 5’RACE, forward and reverse primers respectively. PCR products of 3’RACE (814 bp) and 5’RACE (328 bp) were sequenced and aligned with partial sequence (Fig 1). A full length *Ecmdar* cDNA was assembled after editing overlapping region. A set of gene specific primers for amplification of complete *Ecmdar* CDS were designed from *Ecmdar* cDNA sequence and cloned into pJET 1.2 cloning vector. The cloned fragment (1437bp) was sequenced and later submitted to NCBI (accession number KT230521). The size of full length cDNA is 1676bp, containing an open reading frame of 1437bp, with a 239bp 3’UTR region and encoding a putative 478 amino acid long protein. The size of genomic *mdar* segment is ~5000bp which is similar to size of pea, *Arabidopsis* and rice *mdar* genomic sequences.

### *In-silico* analysis of *Ecmdar* nucleotide and amino acids sequence

#### Homology search

*Ecmdar* CDS was compared with other NCBI database sequences through nBLAST tool, for evaluating its similarity with other nucleotide sequences. *Ecmdar* (1437) showed 99% similarity to *mdar* sequences from *Eleusine coracana* GP45 and GP1 variety and 85%-87% similarity to other monocot sequence. *Ecmdar* CDS encoded a putative 478 amino acid peptide that was submitted to pBLAST for homology and similarity index comparison with other protein sequences. *Ec*MDAR protein showed 94% and 93% similarity with *E*. *coracana* GP45 and GP1 varieties and 79%-84% similarity with other existing monocot MDAR proteins.

#### Phylogenetic relationship

**Fig 2:**
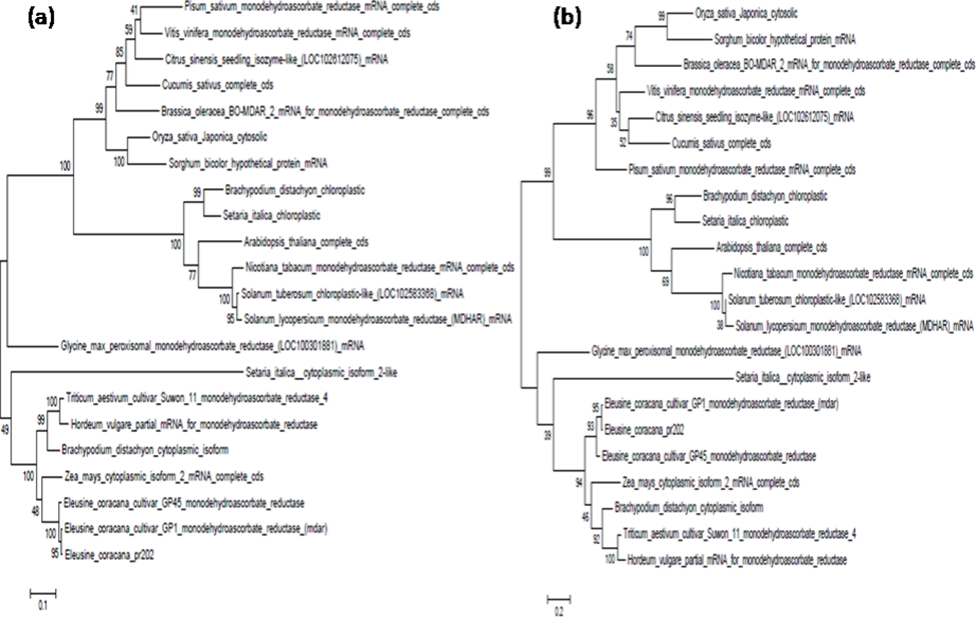
Phylogenetic analysis by MEGA. a) The nucleotide phylogenetic tree with the highest log likelihood (-11769.2602). b)The MDAR protein phylogenetic tree with the highest log likelihood (-5335.6637).

To study the evolutionary relationship of *Ecmdar* nucleotide and amino acids sequence with other monodehydroascorbate reductase sequences, a phylogentic tree was constructed by MEGA6 offline tool (Fig 2). The nucleotide phylogenetic tree was prepared by using maximum likelihood method, based on the Tamura-Nei model [28].The percentage of trees in which the associated taxa clustered together is shown next to the branches. The 1000 bootstrap was used to test the tree. The *Ecmdar* (PR202) is nearest to *E*. *coracana* variety GP1 and GP45, followed by *Zea mays* cytosolic isoform-2 and *Triticum aestivum* peroxisomal isoforms. Protein phylogeny tree was prepared by maximum likelihood method based on Jones-Taylor-Thornton (JTT) model. Phylogeny tree was evaluated by 1000 bootstrap.

#### Conserved domain analysis

Conserved domain search analysis was done to find out the existence of any conserved domains in *Ec*MDAR protein. The analysis showed that *Ec*MDAR contains pyr_redox (pfam00070) and pyr_redox-2 (pfam07992) domains which belongs to pyridine nucleotide-disulphide oxidoreductase family and contain conserved Rossmann fold NAD(P)H^+^ binding domain. Rossmann NAD binding involves several hydrogen-bonds and Van-der-Waals contacts, in particular H-bonding of residues in a turn between the first strand and the subsequent helix of the Rossmann-fold topology. Characteristically, this turn exhibits a consensus binding pattern similar to GXGXXG, in which the first 2 glycine’s participate in NAD(P)-binding, and the third facilitates close packing of the helix with the β-strand. Typically, proteins in this family contain a second domain in addition to the NADH domain, which is responsible for specifically binding a substrate and catalyzing a particular enzymatic reaction. These families include both class I and class II oxido-reductases and alsoNADH oxidases and peroxidases [29]. Pyr_redox domain is 80 amino acid long and is present between amino acid numbers165 to 247. Pyr_redox2 domain containing 301 amino acids and is present between 7 to 325 amino acids.

#### Physiochemical characterization

Physiochemical properties of protein such as theoretical isoelectric point (pI), total number of positive and negative residues, extinction coefficient, instability index, aliphatic index (AI) and grand average hydropathy (GRAVY) provide knowledge about protein stability in cell. Physiochemical properties of *Ec*MDAR are tabulated in Table 2. The deduced molecular weight of the *Ec*MDAR protein is 52.082 KDa and the isoelectric point i.e. the point of pH neutrality is 9.06. PpMDAR isoforms have the optimal activity in the pH range 7.6 to 8.7 [30]. Proteins are stable, compact but least soluble at their isoelectric pH, and can be easily isolated on the basis of their pI by isoelectric focusing.

**Table 2:** Physiochemical properties of *Ec*MDAR predicted by protopram expasy tool.

Properties	Value
Molecular weight	52.08 KDa
pI	9.06
Negative charged residues (Asp + Glu)	48
Positive charged residues (Arg + Lys)	57
Ext. coefficient at 280nm	66155 M^-1^ cm^-1^
Estimated half life	30 hours (mammalian reticulocytes, in vitro). >20 hours (yeast, in vivo). >10 hours (Escherichia coli, in vivo).
Instability index	35.27
Aliphatic index	89.16
Grand average of hydropathicity (GRAVY)	0.007

Instability index analysis showed that *Ec*MDAR is a stable protein. At 280nm, the extinction coefficient of *Ec*MDAR was computed to be 65780 to 66155 M^-1^cm^-1^. Higher amount of Cys, Trp and Tyr leads to a higher extinction coefficient. A quantitative study of protein-protein and protein-ligand interactions in solution can be done using this computed extinction coefficient. The computed instability index (II) of *Ec*MDAR is 35.27.It is accepted that if the instability index of a protein is lower than 40, then the protein is stable in nature.

The estimated half-life of EcMDAR protein under *in-vivo* system, such as *E*. *coli*, was computed to be >10 hours and under *in-vitro* conditions, such as mammalian reticulocytes was calculated to be 30 hours. The half-life of the protein was estimated by ‘N end rule’. The N-end rule is based on observations that the identity of the N-terminal residue(s) of a protein plays an important role in determining its stability *in vivo*. The total number of negative (Aspartic acid + Glutamic acid) and positively charged (Arginine + Lysine) residues were 48 and 57 respectively. Hence, the overall charge on *Ec*MDAR protein is positive. The aliphatic index of *Ec*MDAR protein shows the relative volume of hydrophobic amino acid in protein. The aliphatic index of *Ec*MDAR is 89.16. *Ec*MDAR contains higher percentage of alanine, valine, leucine and isoleucine which contribute to increasing its aliphatic index. A higher aliphatic index indicates better thermo-stability of a protein. The GRAVY value for a protein is calculated as the sum of hydropathy values of all the amino acids, divided by the number of residues in the sequence. The hydrophobic scale of individual amino acids is helpful in prediction of trans-membrane segment(s) of protein. *Ec*MDAR has GRAVY values 0.007 which indicate it is hydrophobic in nature and contain transmembrane segments.

#### Post-translation modification site prediction

**Fig 3:**
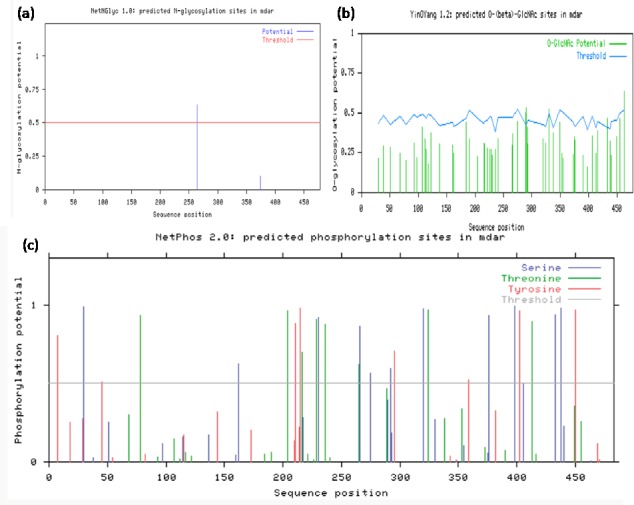
Posttranslation modification site. NetPhos 2.0 and NetNGlyc 1.0 web server were used to computate the potential posttranslation modification sites. Only those amino acids with a score higher than the threshold value are consider for posttranslation modification.

Post translation modification sites such as phosphorylation site & glycosylation site are evaluated for better understanding of protein function and regulation. The function of proteins is frequently modulated by chemical modifications introduced after translation from mRNA. Additionally, these post-translational modifications (PTMs) have been shown to influence the interaction between proteins carrying them [31].Post translation modification site analysis of *Ec*MDAR protein was done by N-glyco server and NetPhos server. NetNGlyc server predicted one potential site (NTSL) for N-linked glycosylation at 264 amino acids position and 5 potential sites for O-linked glycosylation at 289(T), 290(S),330(S),433(S) and 463(S) amino acid positions. NetPhos server predicted twenty seven potential sites for phosphorylation. Eleven sites have serine, eight threonine and eight tyrosine amino acids (Fig 3). The location of Tyr residues in the structure of Pea peroxisomal MDAR reveals that Tyr_345_ is found at 3.3 Å of His313 which is present in the NADP binding site. Site-directed mutagenesis confirmed Tyr_345_ as the primary site of nitration which is responsible for the inhibition of MDAR activity by ONOO^–^. Hence, salt stress induced nitro-oxidative stress causes inhibition of MDAR activity by ONOO^-^ [32]. In *Ec*MDAR, His_317_, Tyr_173_ and Tyr_348_ are present in NADH binding site. Tyr_348_ may be a potential site for nitration induced inhibition of MDAR activity under salt stress.

#### Structural and functional annotation

**Fig 4:**
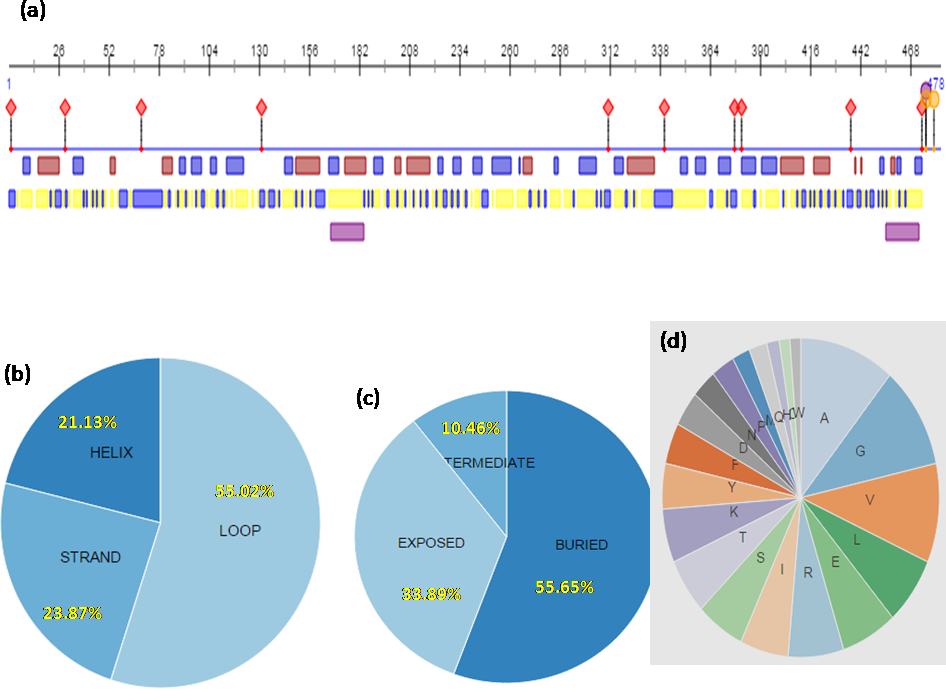
Structural annotation of protein: Prediction of amino acid composition, secondary sturcture, solvent accessibility and transmembrane helix of EcMDAR protein. a) Graphical representation of structural annotation. b) Composition of secondary structure. c) solvent accessibility. d) Amino acid composition.

*Ec*MDAR protein’s structural and functional annotation was done by Predict Protein Online server (www.predictprotein.org). PROFsec predicts secondary structural elements of the protein. Secondary structure analysis of *Ec*MDAR protein suggested that it is a mixed type of protein containing α helix (21.13%), β sheets (23.87%) and β Loop (55.02%). PROFacc predicts solvent accessibility of protein residues for 10 states of relative accessibility and groups the proteins into two states: buried and exposed. *Ec*MDAR protein contains 33.89% exposed region, 55.65% buried region and rest 10.46% intermediate region (Fig 4). Solvent accessibility test of protein concludes that *Ec*MDAR contains high proportion of hydrophobic amino acids which suggests that it may be a membrane bound protein. Further, transmembrane helix prediction of *Ec*MDAR analyzed by TMHMM server 2.0 shows that EcMDAR contains two potential transmembrane helices. First helix was present at 5–24 amino acids position (FVYVILGGGVAAGYVALEFA) and second helix was present at 450–472 amino acids position (VWHATAAVVAAVSIAAVGYWYG), from N terminal of protein.

Disulfide bond formation generally occurs in the endoplasmic reticulum by oxidation. Therefore, disulfide bonds are mostly found in extracellular, secreted and periplasmic proteins, although they can also be formed in cytoplasmic proteins under conditions of oxidative stress. Disulfide bond prediction done by DiANNA 1.1 sever (DiANNA (DiAmino acid Neural Network Application) is a web server that provides two services, namely Cysteine classification prediction and disulfide connectivity prediction [33]. Cysteine classification prediction was done to determine whether each cysteine residue in a protein chain is involved in forming a disulfide bond or not whereas disulfide connectivity prediction was done to identify cysteine pairs likely to form disulfide bonds [34]. DiANNA tool predicted six cysteine residues at 35, 69, 143, 161, 178 and198 amino acid positions in *Ec*MDAR protein. Disulfide bond score prediction of *Ec*MDAR showed that 143–161 (DAENICYLRNI-NAMKSCSGGNA) have highest disulfide prediction score of 0.99 and potential sites of disulfide bond formation are 35–198 (SRGELCIISEE–FPEKHCMARLF), 69–178 (PGFHTCVGAND-YIGMECAAALV) and 143–161 (DAENICYLRNI-NAMKSCSGGNA). Cysteine is not only important to maintain the structural stability of MDAR protein but also has a major role in catalytic action of MDAR protein [35]. It has been reported that C_117_ of chloroplastic MDAR is a conserved amino acid that has a vital role in binding of NADH +H^+^ and maintaining the structural stability by interacting with hydrophilic amino acids [36]. Mutation at C_117_ with A and S has varying degree of effects on catalytic activity of chloroplastic MDAR. Reduction in catalytic constant by C_117_S and C_117_A was found to be 71.1% and 49.5% respectively, as compared to wild-type MDAR. Besides this, the binding affinity of NADH in mutant MDAR was lower as compared to that of wild type MDAR, as indicated by an increase of about 1.5–3 folds in Km values of NADH. However, the mutation at C_117_S had less effect on NADH binding and specific activity of MDAR than that of C_117_A; this has been suggested to be due to the structural similarity between the amino acids Ser and Cys [37]. A cysteine residue at 69 position of EcMDAR was conserved in all the characterized MDAR proteins such as cucumber, pea, spinach and *Physcomitrella patens*. It has been studied that mutation at C_69_S decreases the activity of MDAR in cucumber [38], whereas mutation at Cys_69_ residues of PpMDAR with alanine had little or no influence on PpMDAR2 activity [30]. In *Ec*MDAR, C_117_ is not a conserved amino acid. *Ec*MDAR has Tyr instead of Cys at 117 position. *In silico* analysis of mutation suggested that T_117_C has neutral effect on the structure and function of the protein which confirmed that Tyr_117_ had no specific role in structural stability. On the other hand, mutation at Cys69 with other amino acid, influences the function and structural stability of *Ec*MDAR.

#### 3D computer modeling and its validation

**Fig 5:**
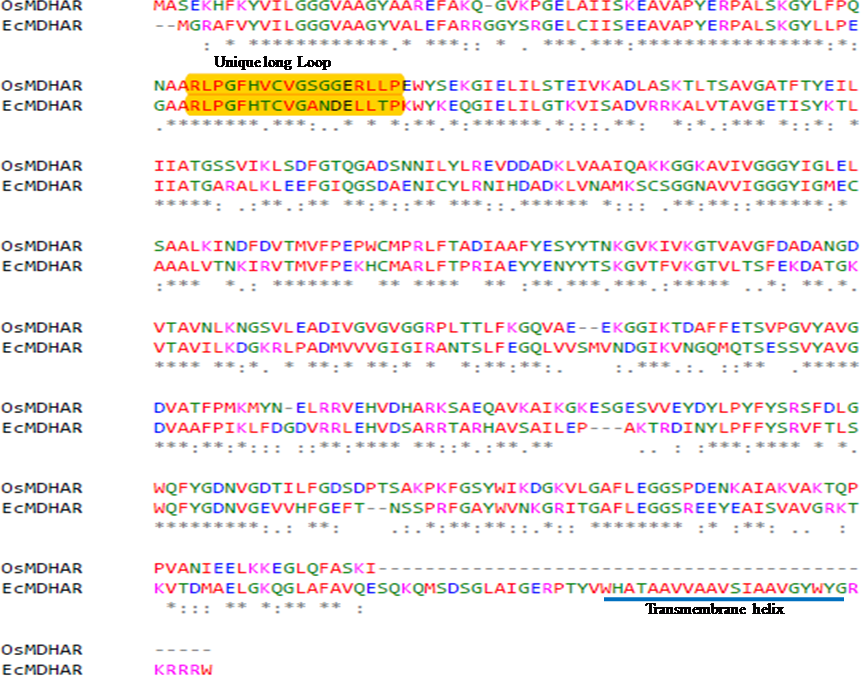
Pairwise sequencs alingment of *Ec*MDAR and OsMDAR amino acid sequence. Sequence is labelled with a unique loop which is conserved in their respective MDAR sequences and extra amino acid sequence in *Ec*MDAR contributed in formation of transmembrane helix.

A three dimensional structure prediction of protein is necessary for evaluating the function and active site residues of protein. Structure and catalytic mechanism of monodehydroascorbate reductase enzyme was studied by [39]. A Crystal structure of *Oryza sativa japonica* monodehydroascorbate reductase enzyme with its cofactor such as FAD, NAD and ascorbic acid was studied by crystallography and submitted in PDB. Three dimensional model of EcMDAR was constructed by swiss model online server based on homology modeling approach. Swiss model is an online server which computes 3 D model on the basis of sequence similarity between query and template. OsMDAR is a 435 amino acid long single chain polypeptide which has 56.7% identity with *Ec*MDAR polypeptide sequence. It covers around 88% area (3-429aa) of *Ec*MDAR protein. *Ec*MDAR protein is a 478 amino acids long polypeptide which contains a 49 amino acid long extra sequence at C-terminal end, which primarily contributes in the formation of transmembrane helix region (Fig 5). The QMEAN score of model is -0.49 and GMQE score is 0.75.

**Fig 6:**
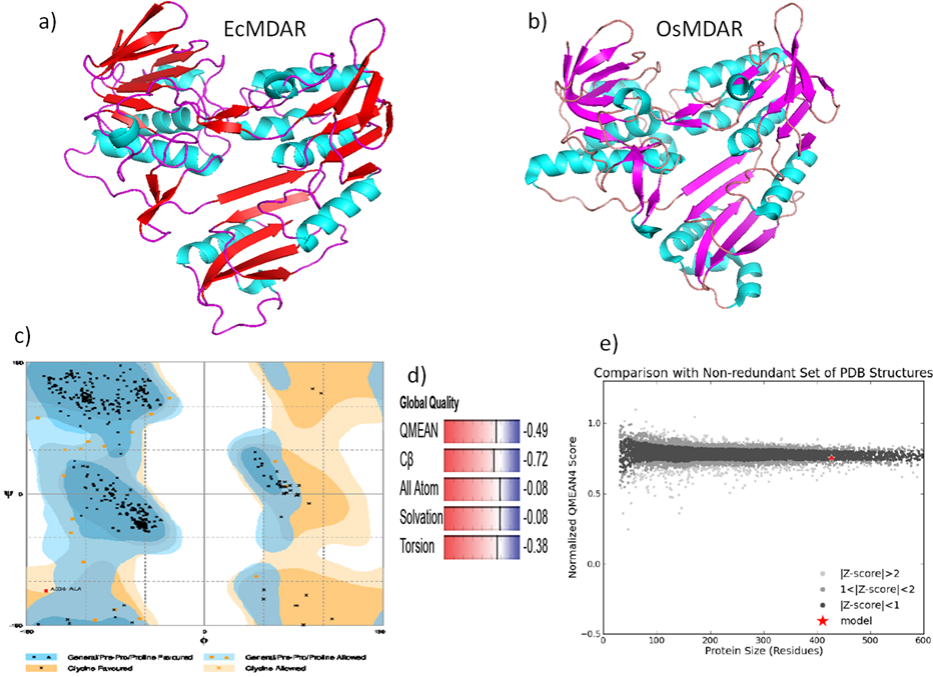
3D modeling of *Ec*MDAR protein. A) 3D model computed by Swiss Model online server using homology alignment approaches. b) OsMDAR crystallography 3D model (PDB– 5jci). c) Ramchandran Plot of *Ec*MDAR depicted > 95.3% residue in favored region. d) Global quality of *Ec*MDAR model based on QMEAN score, Cα and all atoms pairwise distance score of query and template, Solvation energy and torsion angle. e) Z score of *Ec*MDAR predicted model.

3D model of *Ec*MDAR protein contains 21 sheets and 15 helices and 33 loop regions (Fig 6A). *Ec*MDAR also has a unique loop between 63–80 amino acid (Fig 5) which is conserved and present in MDAR of *Arabidopsis thaliana*, *Zea mays*, *Oryza sativa*, *Vitis vinifera*, *Glycine max* and *Brassica rapa* plants, but absent in bacterial oxidoreductase enzymes. QMEAN score is the estimation of global and local model quality. It is based on four structural features of model: 1) torsion angle based local geometry, 2) two pairwise distance assessment for all atomic interaction, 3) distance assessment for beta carbon interaction and 4) solvation potential. QMEAN score of the model directly relates with Z score which relates the global QMEAN value of model with the Z score of high resolution X-ray structure (PDB submitted) (Fig 6D and 6E). GMQE (Global Model Quality Estimation) is also a quality estimation based on target template alignment. GMQE explains the accuracy of model on the basis of alignment and template. OsMDAR contain 13 helices, 25 sheets, 5 turns and 34 loops in tertiary structure (Fig 6B). Validation of 3D model of *Ec*MDAR was done by Errate and Verify 3D online tools. *Ec*MDAR 3D model passed the verify 3D with 100% as100% of its residues had an average 3D-1D score > 0.2. Errate is used to verify the crystallographic structure of proteins [40]. In Errate, error values describe the statistics of non-bonded atomic interactions. The overall quality of *Ec*MDAR model in Errate was 96.359 which indicates, that this model has good and high resolution structure. Ramchandran plot analysis by RAMPAGE showed that 95.5% residues were in favored regions and 4% in allowed regions (Fig 6C).

#### Active site prediction

**Fig 7:**
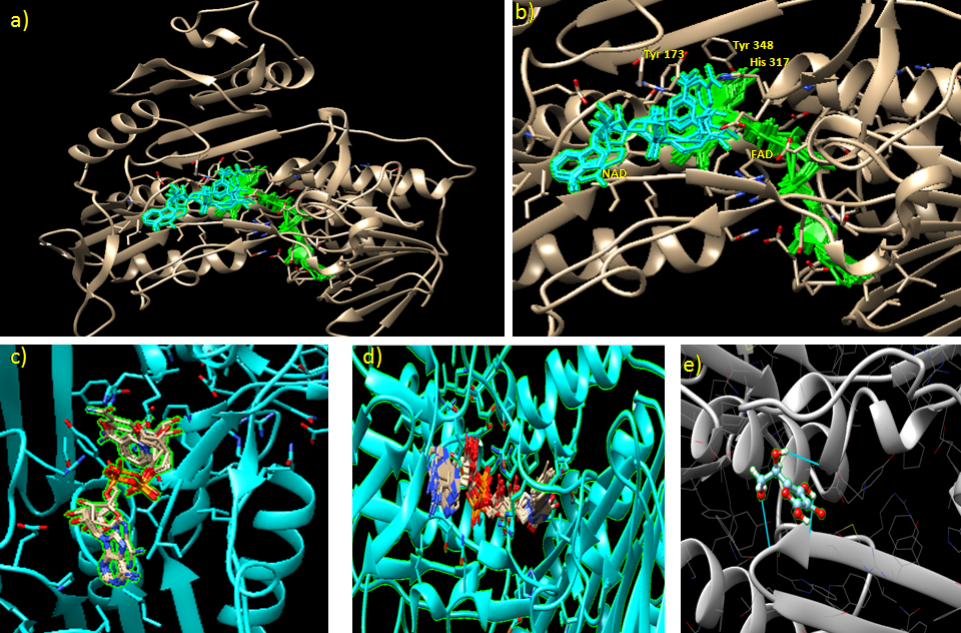
Active site prediction by 3Dligandsite online tool. a) Ligand binding at active site. b) Amino acids (Tyr 348, Tyr_173_ and His_317_) at active site interacting with both NAD and FAD. c) binding domain of NAD. d) binding domain of FAD. e) binding domain of ascorbic acid and hydrogen bond interaction of ascorbic acids with Lys, phe and Arg residues.

Active site prediction of *Ec*MDAR was done by ligand 3D site online tool. Active site was predicted by 1) superimposing ligand binding site of the known structure with query protein 3D structure and 2) mapping the conserved residues of ligand binding sites in query protein [41]. *Ec*MDAR active sites contain four antiparallel sheets, three loops and four helices. Active site of *Ec*MDAR contain Glu_39_, Glu_40_, Glu_46_, Arg_47_, Pro_48_, Lys_52_, Thr_122_, Asn_147_, Tyr_173_, Pro_194_, Glu_195_, Arg_201_, Ile_261_, Asn_264_, Asp_299_, Glu_316_, His_317_, Arg_322_, Phe_347_ and Tyr_348_ (Fig 7A). These amino acids interact with ligands and stabilize them in active site *via* hydrogen bond and Van-der-Waals interaction. In *Os*MDAR, two antiparallel loops, one parallel loop and four helices contribute in the formation of active site. Tyr_348_ is conserved amino acid which participates in electron transfer reaction between FAD and Ascorbic acid. Tyr_348_ is catalytic residues of MDAR. During the oxidation reaction, initially two electrons are transferred from NAD(P)H in the hydride transfer to FAD in OsMDAR followed by sequentially transfer of two electrons from FADH_2_ to Tyr_349_, resulting in a flavin semi quinone reaction intermediate. The electrons are then transferred from the Tyr_349_ to the MDHA radical, generating two molecules of AsA [39].

3D model of *Ec*MDAR protein was docked with Ascorbate, NAD and FAD ligand to predict the amino acids which facilitate the binding of these ligands at crevices of active site. Asp_279_, Arg_201_, Ile_259_ and Glu_195_ interact with FAD and form hydrogen bond. Asp_279_ interacts with HO12 of ribose moiety, Ile_259_ and Glu_195_ with 9NH and 3NH of adenine respectively. Arg_201_ stabilizes FAD by forming two hydrogen bonds with O5 of phosphate group and HN7 of adenine. In *Os*MDAR, Gly_13_, Gly_15_, Ala_122_, Thr_123_, Gly_297_, and Ala_319_ participate in Van-der-Waals interaction with FAD. Similarly Gly_12_, Gly_13_, Thr_122_, Ala_124_, Gly_298_ and Ala_321_ are present at FAD binding domain which might be involved in Van-der-Waals interactions (Fig 7D). NAD binding domain is made up of α helix, loop and β sheets. It is surrounded by 3 helices, two antiparallel and 4 parallel β sheets. The nicotinamide ring of NAD is sandwiched between Tyr_173_ and iso-alloxazine ring of FAD. His_317_ also interacts with nicotinamide ring of NAD (Fig 7B). NAD forms hydrogen bond with Glu_177_, Glu_316_, Arg_146_, Gly_171_ and Tyr_348_. In OsMDAR, Glu_178_ residue participates in hydrogen bond interaction with the nicotinamide ring as well as in Van-der-Waals interactions. The OE2 atom of Glu_178_ forms a hydrogen bond with the N7N atom of the nicotinamide ring. The NH2 atom of Arg_202_ forms hydrogen bond with the O2D of the ribose and the O2A of the phosphate group, respectively. An additional hydrogen bond was formed between the OE1 atom of Glu314 and the O3D atom of ribose [39].

The ascorbic acid is bound within the cavity of NAD domain. Ascorbic acid forms three hydrogen bonds with Lys_52_ (LysO2:H5 Asa), Phe_352_ (Phe HN:O6 Asa) and Arg_350_ (O:H2) with binding energy ΔG– 6.29 Kcal (Fig 7E). In OsMDAR Gly_72_, Arg_320_ and Arg_351_ are close to AsA molecule but only Arg_320_ forms hydrogen bond with ascorbic acid. Mutation analysis showed that Arg_320_ is important for binding of substrate to the enzyme [39].

The sequence alignment of *Ec*MDAR with other peroxisomal MDAR showed that it has a consensus region at C terminal (WYGRKRRRW) which is present in peroxisomal MDAR protein of other monocots and dicots also (Table 3). Although *Ec*MDAR protein also has high similarity with cytosolic MDAR of other monocots, apart from peroxisomal MDAR, but due to the presence of peroxisomal specific consensus amino acid signal (WYGRKRRRW), *Ec*MDAR is predicted as membrane bound peroxisomal isoform of monodehydroascorbate reductase.

**Table 3:** C terminal consensus sequence present in putative peroxisomal MDAR protein of monocot and dicots.

Organisms	ID & Reference	C- terminal region of peroxisomal MDAR polypeptide
*Eleusine coracana* (478aa)	AJA30318.1 (Negi et al., 2014)	TKVTDMAELGKQGLAFAVQESQKQMSDSGLAIGERPTYVWHATAAVVAAVSIAAVGYWYGRKRRRW
*Arabidopsis thaliana*(488aa)	NP_189420.1 (Salanoubat et al., 2017)	GFAHTVVSQQKVPEVKDIPSAEMVKQSASVVMIKKPLYVWHAATGVVVAASVAAFAFWYGRRRRRW
*Triticum aestivum*(476 aa)	AFU52947.1 (Feng et al., 2014)	TKVLDMPELERQGLAFAIQESKKDVPDSGVTLGEKPTFVWYATAGVVAAVSISAFGYWYGRKRRRW
*Oryza sativa Japonica*(476aa)	NP_001047875.1 (Tanaka et al 2010)	AKVINIAELEKQGLMFAIQESQKDLPDGGLALGEKPTYVWHATAGVIAAASIAAFGYWYGRKRRRW
*Vigna angularis*(478aa)	XP_017419506.1 (This record was predicted through automated computational analysis of (NC_030639.1) by NCBI in 2016.	KVTVKDMTQLERDGLEFAFQISQQAMPEVGVIVERPMYAWHATAGVAVALSISAFAYWYGRRRRIW
*Daucus carota*(478aa)	XP_017243507.1 (This record was predicted through automated computational analysis of NC_030384.1) by NCBI in 2016).	LTELERQGLSFALTLKHDPLPTPTVNVRIPDIVVEKPIYAWHATAGVIVAASVAAFAFWYGRRRRRW
*Cucumis melo*(478aa)	XP_008458779.1 (This record was predicted through automated computational analysis of (NW_007546315.1) by NCBI in 2016).	LESRGLSYAMAISREPPPSQVTNVDVSGPSLVLEKPMYRWHATAGVILAGSVAAFAYWYGRRRRRW
*Capsicum annuum (480aa)*	XP_016561566.1 (This record was predicted through automated computational analysis of (NC_029978.1) by NCBI in 2016).	ETQGLEFALALSRQAEPTEAVVVSSGSGSTIVTEKPLHAWHATAGVILAASIAAFAYLYGRRRRRR
*Arachis duranensis* (480aa)	XP_015971600.1 (This record was predicted through automated computational analysis of (NW_015490735.1) by NCBI in 2016).	LERQGLTFAVTVSQKQLSSPPQIQVTSSTDLVLEKPLYAWHATAGVILAASVAAFAYYYGKKRRRW
*Citrus sinensis (479aa)*	XP_006470310.1 (This record was predicted through automated computational analysis of (NW_006256945.1)byNCBI in 2016).	ELETQGLGFALAVSQKPLPSTPVDGKTVPGLVLGKSLYPLHATAGVILAASIAAFAYWYGRRRRRW
*Solanum tuberosum* (481aa)	XP_006338572.1 (This record was predicted through automated computational analysis of (NW_006238930.1) by NCBI in 2016).	ETQGLGFALTLSDKPEPSEAVVVGSGSGSTLATEKPLHVWHATAGVILAASIAAFAYWYGRRRRRW

#### *In silico* analysis of *Cis* regulatory elements of *Oryza sativa mdar* promoter sequence

**Fig 8:**
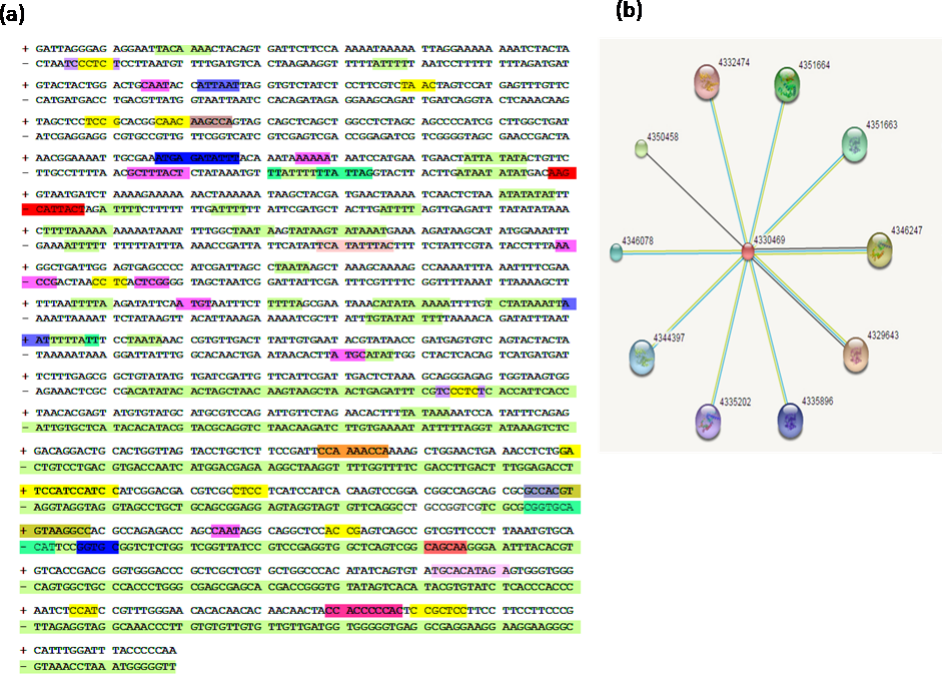
*In-silico* promoter prediction and protein-protein interaction of MDAR. a) Cis regulatory elements of the promoter. b) String based analysis of MDAR protein.

On the basis of sequence similarity index, *Oryza sativa mdar* genomic sequence (gi|297610002) comprising of 3948bp was found to have the closest match to the *Ecmdar* sequence. Therefore *Osmdar*5’ genomic sequence upstream from transcription start site, was retrieved from NCBI database and submitted to promoter prediction 2.0 tool, for prediction of potential promoter region. The 1140bp region (score-1.28) was predicted as highly likely transcription start site. For the analysis of promoter *cis*-regulatory element, 1140bp sequence was subjected to ‘PLACE’ online server, for computing the *cis-*regulatory element(s). The PLACE tool predicted several universal motifs in the promoter region such as CAAT box, TATA box, AT-1 motif, 3AF1 binding, I-box, LAMP element, G-box, ABRE, A-box, TGA box and TCCT box (Fig 8). The highest score motifs were TATA box (core promoter element at -30 of transcription start site), A-box (sequence conserved in alpha-amylase promoters), AT1-motif,3-AF1 binding site, I-box, LAMP-element *etc*. (all part of light responsive element),and WUN-motif (wound-responsive element) (Table 4). A high number of light responsive element presence indicated that MDAR activity is strongly influenced by the presence of light. [27] has reported that the differential activity of peroxisomal and cytosol MDAR isoforms in tomato could be due to the difference in ascorbate level in response to light. Promoter analysis of tomato and pea MDAR genes has suggested that light responsive elements are frequently present in promoter region of MDAR gene and influence the expression of MDAR [42,43]. The transcript levels of ascorbate recycling genes, MDAR and DHAR, were gradually repressed in response to dark treatment in *Acerola* leaves [44].

**Table 4:** *Cis* regulatory elements predicted in *Osmdar* promoter.

Motifs	Sequence	Function
3-AF1 binding site	AAGAGATATTT	light responsive element
A-box	TATCCATCCATCC	sequence conserved in alpha-amylase promoters
ABRE	ACGTGGC	cis-acting element involved in the abscisic acid responsiveness
ARE	TGGTTT	cis-acting regulatory element essential for the anaerobic induction
AT1-motif	AATTATTTTTTATT	part of a light responsive module
Box 4	ATTAAT	part of a conserved DNA module involved in light responsiveness
Box III	CATTTACACT	protein binding site
CAAT-box	CAAT	common cis-acting element in promoter and enhancer regions
CCGTCC-box	CCGTCC	cis-acting regulatory element related to meristem specific activation
G-Box	CACGTG	cis-acting regulatory element involved in light responsiveness
I-box	GTATAAGGCC	part of a light responsive element
LAMP-element	CCAAAACCA	part of a light responsive element
WUN-motif	TCATTACGAA	wound-responsive element
box II	TCCACGTGGC	part of a light responsive element
TGA-element	AACGAC	auxin-responsive element
TATA-box	TACAAAA, TATA, TTTTA, TATATAA	core promoter element around -30 of transcription start
Sp1	CC(G/A)CCC	light responsive element
TCCC-motif	TCTCCCT	part of a light responsive element

#### Interacting protein partner analysis

STRING based analysis of MDAR protein predicted the functionally interacting partner network. STRING is a biological database of known and predicted protein-protein interactions. Subjecting the *Ec*MDAR protein sequence to STRING analysis, showed high similarity with *Oryza sativa (japonica)* MDAR proteins ID 4330469 (476aa, bit score -829 and E value—0) and ID 4330468 (479aa, bit score-598 and E value—0). The STRING based analysis of *Ec*MDAR protein predicted different ascorbate peroxidase isoforms as the functional partners (Fig 8B).

### Bacterial expression of *Ec*MDAR protein

In order to express the recombinant *Ec*MDAR protein, pET23b(+) vector was used for heterologous expression of *Ecmdar* CDS in *E*. *coli* BL21-DE3 cells. *E*. *coli* cells were grown at 37°C until the O.D._600_ of cells reached 0.5 units. At 0.5 OD, IPTG was added for inducing the expression of *Ec*MDAR. After cell lysis the protein was isolated from the pellet fraction. It was observed that highest expression of *Ec*MDAR occurred in the pellet fraction, when 0.3mM IPTG was used for induction and cells were grown at 22°C for overnight with vigorous shaking. In AIM induction media, protein was expressed in the soluble form. The SDS-PAGE analysis showed a ~52 KDa protein band, in pellet fraction of transformed *E*. *coli* cells that perfectly corroborated with our results (S2 Fig).

### Expression analysis of *mdar* under stress

#### Water deficit stress

**Fig 9:**
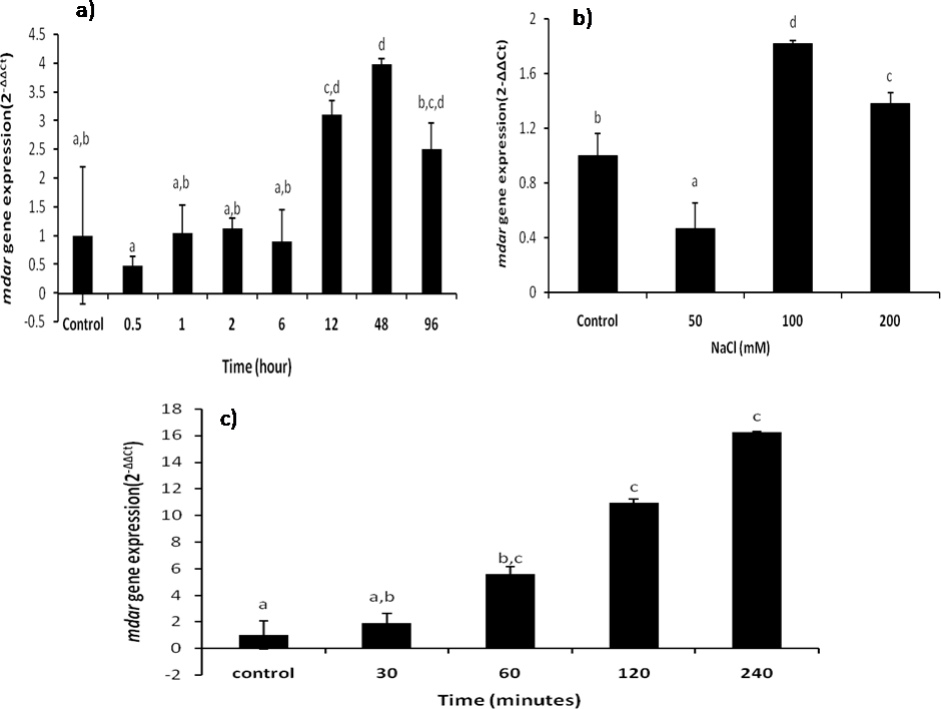
*Ecmdar* gene expression studies. Transcript accumulation of Ec*mdar* under a) PEG mediated drought stress at different time intrervals (0.5, 1, 2, 6, 12, 48 and 96 hrs). b) Salt stress at 0, 50,100 and 200mM concentration. c) UV-C stress at different time intrerval (30, 60,120 and 240min. Different letters indicate means that differ significantly (P < 0.05).

Quantitative real time PCR analysis of *mdar* gene in finger millet seedlings, grown under PEG mediated water deficit stress, at different time intervals, was done to evaluate the temporal expression of *Ecmdar*. The gene expression pattern of *mdar* given in terms of 2^-ΔΔCt^ method appeared non-significant till 6 hours (Fig 9A). At 12 hours, significant *mdar* expression was induced, which continued till 48 hours. After 48 hours, a reduction in *mdar* transcript was recorded at 96 hours. These observations suggest that *mdar* could be an early responsive gene. The present results are also supported by previous reports of *mdar* gene expression, wherein differential expression pattern of *mdar* under various stress conditions was reported by [16]. In another study, [45] reported the transcript levels of AsA-GsH pathway genes in wheat seedlings under PEG mediated stress and in response to ABA. They have reported an increased transcript levels of several antioxidants genes at different time points, such as *gr* at day1, *dhar* at day 4, *mdar* at day1, and *gs* at day4 [45].

#### Salt stress

The direct effects of imposition of NaCl induced salt stress include induction of ion toxicity and osmotic imbalances; while the indirect stress effects include the generation of reactive oxygen species [46]. Under increasing level of salt stress, differential *mdar* expression pattern was recorded. A reduction in *mdar* transcript level (0.53 fold) was recorded at 50mM NaCl treatment, while exposure to 100mM NaCl stress increased the *mdar* gene expression by 0.82 folds, as compared to the control seedlings. At 200mM salt stress, *mdar* expression was higher than the control, but was less than that observed at 100mM NaCl concentration (Fig 9B).

Plant’s response to salt stress is a complex phenomenon and involves a number of gene families which response to ionic and osmotic stress components. Plants adapt to salt stress by regulating ionic and osmotic homeostasis. The antioxidant system response varies from plant to plant under oxidative stress and dependents on the magnitude and exposure time of stress [47]. [48] reported induced expression of *mdar*-1 and *mdar*-3 isoforms under salt and osmotic stress, whereas *mdar*-2 isoform expression remained unaltered, in *Physcomitrella patens*. The *mdar* gene is significantly up-regulated and, accordingly, the pool of reduced ascorbic acid was found to be increased in trichoderma treated plants that resulted in increased salt stress tolerance [49]. However, with respect to different enzymatic isoforms of MDAR, there are reports indicating a decrease in MDAR under salt stress, indicating a complex manner of regulation.

#### Ultra-violet radiation stress

UV-C radiations have been reported to induce reactive oxygen species generation in plants [50]. At very low magnitude of exposure to UV light, induction of reactive oxygen species starts, that acts as signaling molecule to induce the activity of antioxidant enzymes. In the current experiments UV-C exposure was given to the *Eleusine coracana* seedlings for different time intervals (0, 30, 60, 120 and 240 min). The accumulation of *mdar* transcript upon UV-C exposure increased with UV-C exposure time. At 30, 60, 120 and 240 minutes a continuous increase in *mdar* transcript was recorded, as compared to the control seedlings. The increase in *Ecmdar* transcript was 0.89, 4.59, 9.96 & 15.27 folds at 30, 60, 120 and 240 minutes, respectively (Fig 9C). In *Arabidopsis thaliana*, induction of *mdar* activity has also been reported under UV stress by [51]. [16] have also reported induction of *mdar* gene expression within 15min of treatment. These results clearly indicate that *mdar* gene acts as an early responsive gene.

UV-C radiations are highly ionizing radiations and promote the photo-reduction of di-oxygen, which leads to generation of superoxide radicals. Monodehydroascorbate reductase has an important function in chloroplasts, wherein it regenerates ascorbate from monodehydroascorbate (MDHA). However, in absence of MDHA, the same enzyme catalyzes the photo-reduction of di-oxygen to superoxide radical [38]. This alternative activity of MDAR could lead to the production of highly toxic superoxide radicals that can further oxidize various bio-molecules. Therefore, the expression of *mdar* is highly regulated in plants, and makes it a potential candidate for the first line anti-oxidant defense enzyme.

[52] reported that in *Vitis vinifera*, the *mdar* transcript was down-regulated after 24 hours exposure to high light stress. Indicating the extreme exposure to stress leads to down-regulation of the mdar transcript, as is recorded in our experiments also. Further, [53] reported that in cotton roots the expression of *mdar* gene decreased under salt stress, possibly indicating that the expression of *mdar* is regulated by light. Thus, from the above discussion it is amply clear that *mdar* is a stress responsive gene and plays a critical role in plant antioxidant defense.

### Functional validation of cloned *Ecmdar* through *In-planta* transformation of *Arabidopsis thaliana*

*In planta* transformation has advantages over tissue culture based methods, as it reduces the number of steps required to develop transgenics and undesirable effects on phenotype and genotype of transformed plants. In the current studies floral dip in-plant transformation method was used to develop transgenic *Arabidopsis* plants carrying *Ecmdar* gene construct. Infected plants were allowed to grow till maturity and seeds were collected. These seed were then used for selection of *Ecmdar* transformed lines.

#### Screening of transformed plants

Primary screening of transformed plants was done through BASTA selection. Two hundred mature seeds, obtained from floral dip treated plants, were sown in pots and kept at 4°C in dark for 7 days for vernalization. Thereafter, the plants were grown under controlled conditions in a green house. At 4–6 true leaf stage, *Arabidopsis* seedlings were subjected to BASTA treatment. BASTA (ammonium salt of glufosinate) was sprayed three times on alternate days. Untransformed seedlings were burnt due to accumulation of ammonia in the seedlings, whereas the transformed seedlings grew well and remained green due to the presence and expression of BASTA resistant (*blpR*) gene (S3 Fig). Glufosinate or phosphinothricin is an analogue of glutamate which irreversibly binds to glutamine synthase enzyme and inhibits the synthesis of glutamine from glutamate and ammonia. Accumulation of ammonia inside the cells leads to toxicity and cell death. BASTA is considered as a stable selection marker and in our experiments it showed little false positive results.

#### PCR confirmation of transformed plants

Putative transformed plants, selected on the basis of BASTA resistance, were tested for the presence of *Ecmdar* gene construct through PCR. The primer set used for selecting the transformed plants were designed from the flanking regions of *mdar*, containing the recombination sites. Plasmid carrying the *Ecmdar* gene was taken as positive control, whereas wild type *Arabidopsis* genomic DNA was taken as the negative control. All the BASTA resistant plant showed the presence of a distinct band of ~1.5 kb *Ecmdar* CDS, whereas the wild type *Arabidopsis thaliana* (negative control) did not reported any band (S3 Fig). The results prove that BASTA resistance gene is a good selectable marker having 100% efficiency for true to type selection. BASTA resistant gene is less harmful as compared to antibiotic selection marker gene and is equally effective for screening of putative transgenics.

The transformation efficiency of the floral dip method was calculated to be 1.90% as 19 transgenic plants were screened, out of 1000 seeds in BASTA screening. [54]used vacuum infiltration transformation strategy in the bolting plants of various heights and reported the transformation efficiency in the range of 0.1–0.75 percent; and by floral dip method an average percent transformation efficiency of 0.5–3% was reported.

Transgenic plants confirmed through BASTA screening as well as PCR conformation, were further grown in pots under green-house condition. At flowering stage, inflorescence of plants was covered with translucent envelopes to facilitate self-fertilization. After maturation, seeds of T_1_ plants were collected and sowed in pot soil.

#### Expression analysis of *Ecmdar* in transgenic *Arabidopsis thaliana*

**Fig 10:**
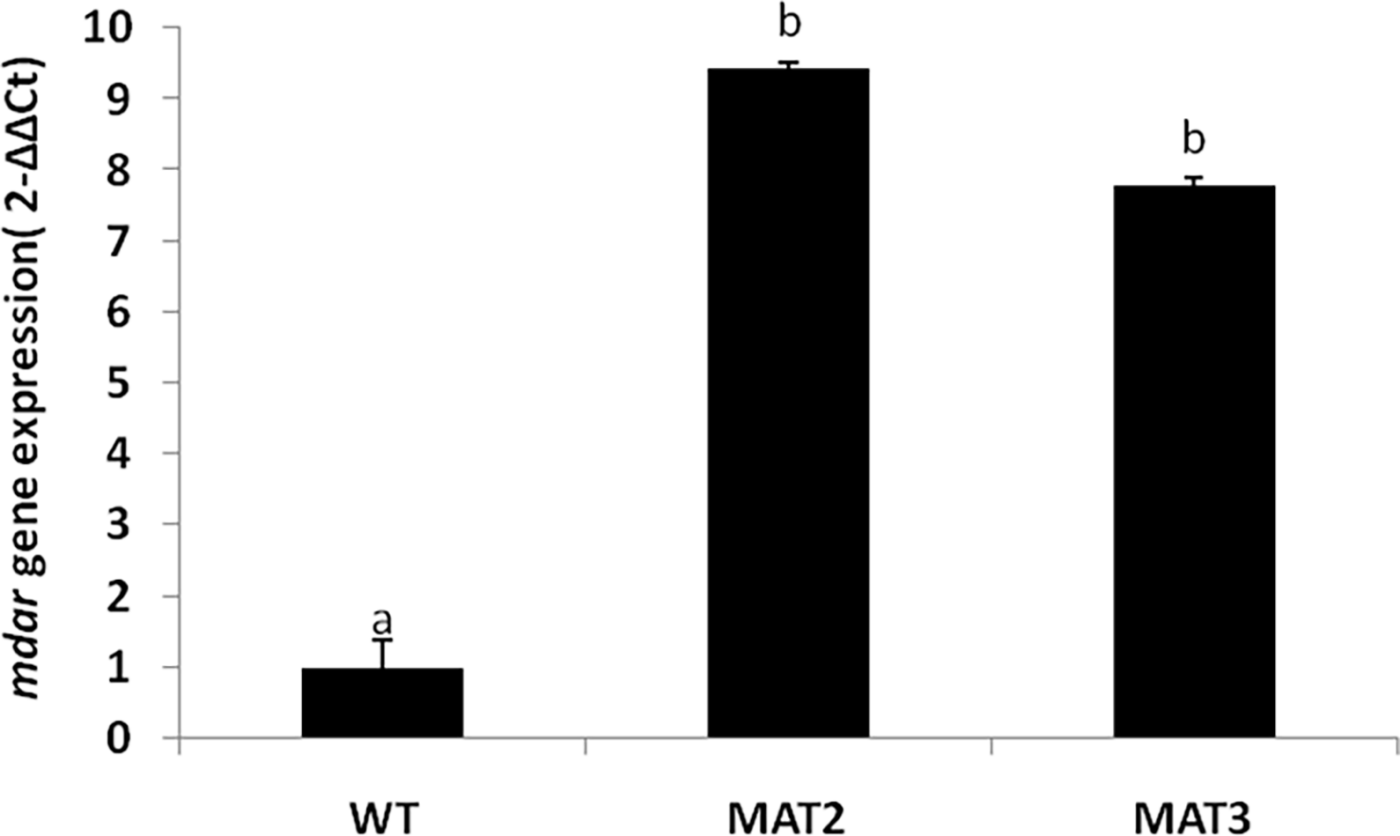
qPCR analysis of *Ecmdar* expression in wild type and two transgenic *Arabidopsis thaliana* lines. Data are presented as Mean±SE. Different letters indicate means that differ significantly (P < 0.05).

Quantitative real time PCR analysis was done to characterize the expression of *Ecmdar* gene in transgenic plants. Monodehydroascorbate reductase transcript level was recorded to be 8.4 fold and 6.7 fold higher in transgenic lines MAT2 and MAT3, respectively as compared to the wild type plants (Fig 10), thereby confirming the successful expression of the cloned *Ecmdar*.

#### Analysis of *Ecmdar* over-expression in *Arabidopsis thaliana*

Analysis of the transformed plants was done to elucidate the effect of *Ecmdar* over-expression on the growth profile and stress tolerance potential of *Arabidopsis thaliana*. *Arabidopsis thaliana* transgenic lines, validated for the over-expression of *Ecmdar*, were used to study the morphological and biochemical changes, if any and their impact on the stress tolerance profile.

#### Growth profile

Transgenic plants did not show any significant differences in the growth pattern and plant morphology, as compared to the wild type plants. The transgenic plants also did not show any significant difference in percent germination with respect to the wild type plants.

**Fig 11:**
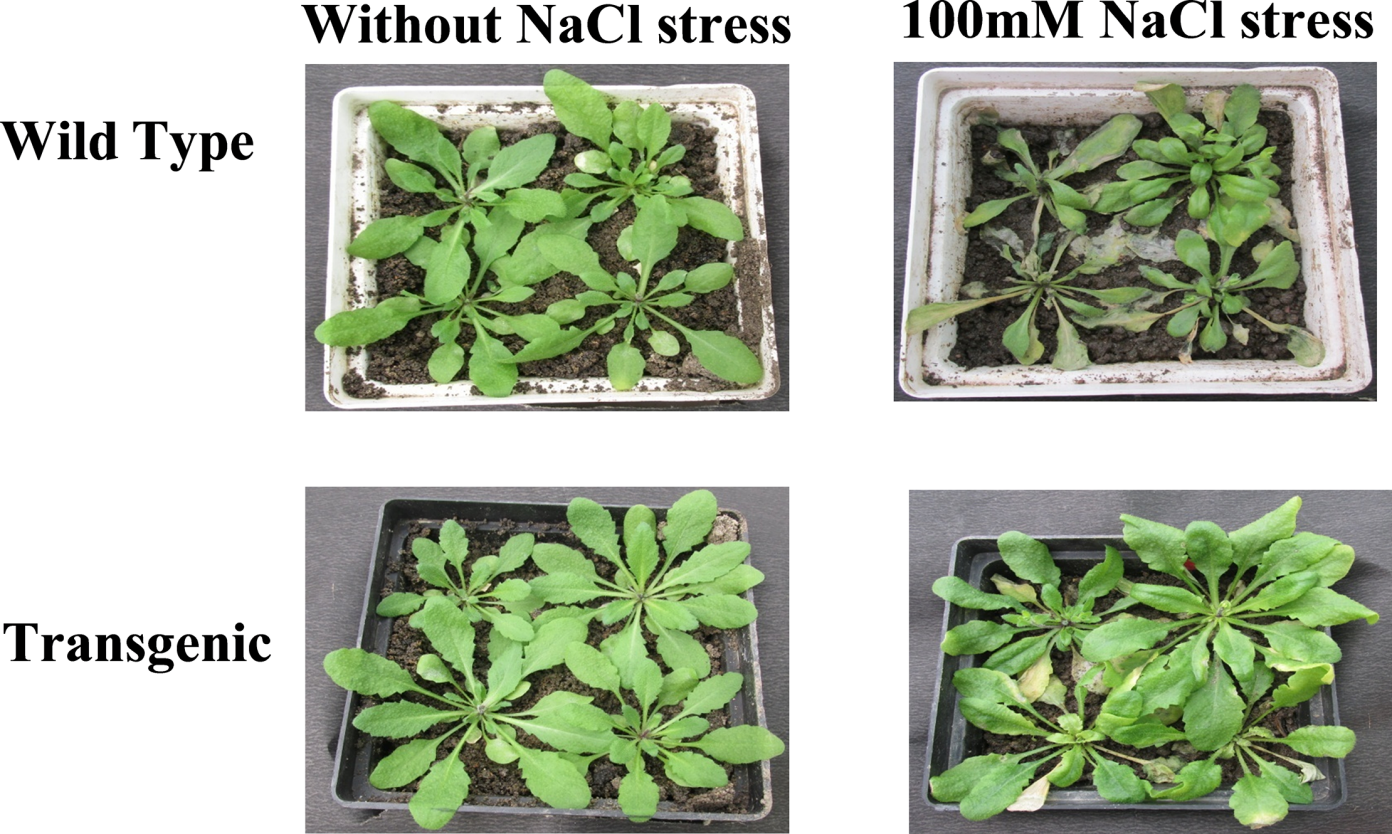
Phenotypic evaluation of *Arabidopsis thaliana* under control and 100mM NaCl-stress conditions.

Under 100mM NaCl stress, transgenic plants recorded better growth as compared to the wild type plants (Fig 11). In presence of 100mM NaCl stress provided for 5 days, wild type plants had shrunken and pale yellowish leaves, whereas transgenic plants had healthy leaves. Thus transgenic *Arabidopsis* plants showed increased salt-stress tolerance as compared to the wild type plants. Morphological observations of transgenic and wild type plants under salt-stress suggest that overexpression of *Ecmdar* positively influences stress tolerance potential of the transgenic plants.

#### Biochemical marker analysis

**Fig 12:**
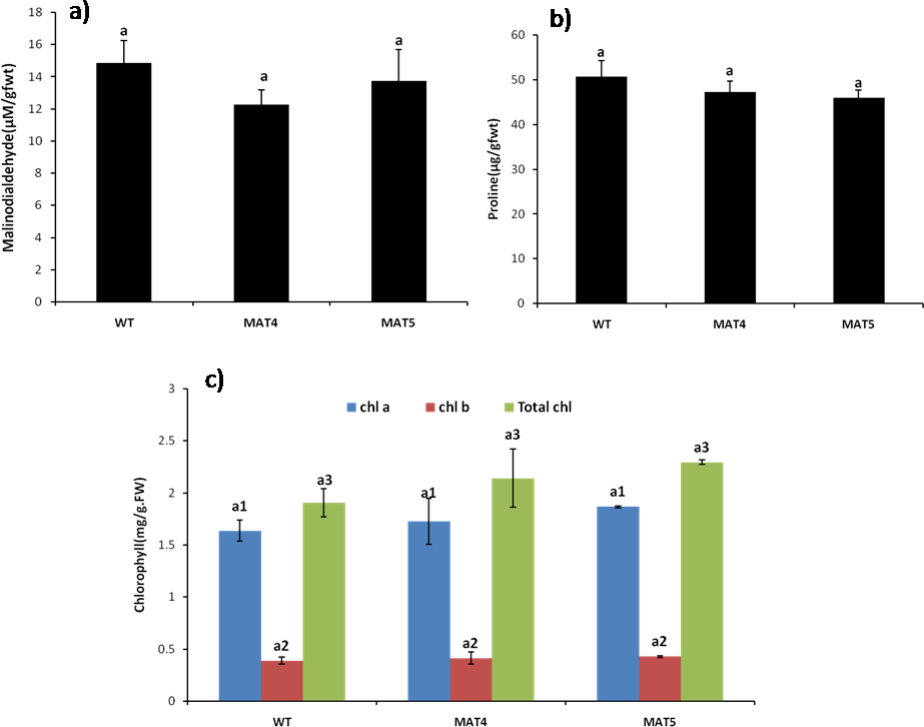
Biochemical marker analysis in wild type (WT) and transgenic lines (MAT4 & MAT5) under optimal growth conditions. a) malondialdehyde content b) proline content c) chlorophyll content. Vertical bars represent Mean±SE. Different letters indicate means that differ significantly (P < 0.05).

Malondialdehyde content was assayed in the wild type as well as transgenic plants, so as to analyze the degree of lipid peroxidation reaction, induced by reactive oxygen species (ROS). Malondialdehyde is produced by oxidation of fatty acyl chain of lipid membrane by free oxygen radicals *i*.*e*.it is an indicator of oxidative stress. The transgenic lines had lower MDA content as compared to the wild type plants. Transgenic lines, MAT4 and MAT5, recorded 17% and 7% lower MDA content as compared to wild type plants (Fig 12A). Reduction of MDA content in transgenic lines indicates that over-expression of monodehydroascorbate reductase enzyme effectively reduces the ROS accumulation. Electrolyte leakage analysis, an indicator of membrane damage by oxidative stress, also indicated that transgenic lines MAT4 (31%) and MAT5 (29%) had significantly lower membrane electrolyte leakage as compared to the wild type plants.

Transgenic plants recorded no statistically significant difference in the proline content vis-à-vis the wild type plants, when grown under optimal conditions (Fig 12B). Proline has been widely reported to be a marker for induction of stress, as it is known to accumulate in plants exposed to a variety of internal as well as external stresses. No statistically significant alteration in proline content in transgenic lines as compared to wild type plants, indicates that over-expression of *Ecmdar* did not have any negative impact on the health (growth) of the transgenic plants.

Chlorophyll content is an essential marker for growth in plants. Chlorophyll biosynthesis is inhibited by several factors including the oxidative damage occurring due to increased amount of ROS [55]. In the present study, chlorophyll content of transgenic *Arabidopsis thaliana* lines MAT4 (12%) and MAT5 (20%) were higher with respect to the wild type plants (Fig 12C). This indicates that optimization of ROS detoxification mechanism in transgenic plants exerted a positive impact by augmenting the bio-synthesis or reducing the degradation of chlorophyll molecules. As chloroplasts are a major site for ROS production, any mechanism strengthening the ROS detoxification exerts a positive impact.

#### Functional validation of transgenic plants under salinity stress

*Arabidopsis thaliana* transgenic lines over-expressing *Ecmdar* (MAT2) as well as wild type plants, grown in green house, were subjected to 100mM NaCl induced salinity stress for 5 days. On 6^th^ day, leaf samples were used for stress marker analysis, to validate the effect of over-expression of *Ecmdar* on plant stress tolerance potential.

**Fig 13:**
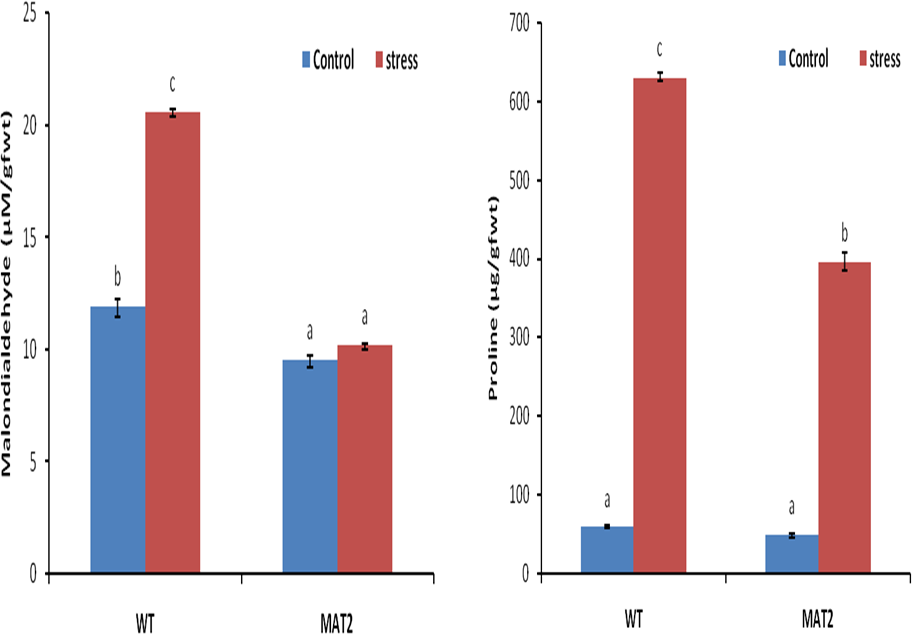
Malondialdehyde and Proline content in wild type (WT) and transgenic (MAT2) *Arabidopsis thaliana* line under 100mM NaCl stress. Vertical bars represent Mean±SE. Different letters indicate means that differ significantly (P < 0.05).

Malondialdehyde content: Wild type plants recorded a 73% increase in MDA content under 100mM salt stress as compared to control conditions (Fig 13). However in the transgenic lines, MDA content was significantly lower than the MDA content in wild type plants, under 100mM NaCl induced stress. The transgenic lines had non-significant induction (6%) of MDA under 100mM salt stress, indicating that MDAR is effectively regulating the accumulation of ROS. Under salt stress, the MDA content in the transgenic line was 50% lower than wild type plant. This indicates that the extent of lipid peroxidation in the transgenic line was significantly lower as compared to the wild type plants. Under stress conditions, plants exhibit higher electrolyte leakage due to increased production and accumulation of ROS. However, under the imposed salt stress, transgenic plants showed statistically non-significant increase in electrolyte leakage, whereas wild type plants recorded a 1.4 fold increase in electrolyte leakage. These results clearly indicate that overexpression of *Ecmdar* effectively reduced the ROS accumulation and consequent degradation of the membrane lipids in transgenic *Arabidopsis thaliana* lines. [17] have reported lower MDA content in transgenic *Arabidopsis thaliana* plants, overexpressing *Brmdar*, as compared to wild type plants under chilling stress.

Proline content: Proline content in transgenic *Arabidopsis* lines was lower than the wild type plants, under control as well as salinity stress conditions. Under 100mM NaCl induced salt stress, proline accumulation was recorded in both wild type and transgenic lines, but wild type plants had significantly higher proline accumulation as compared to the transgenic line (Fig 13). The transgenic line had 38% lower stress induced proline accumulation as compared to wild type plants.

Proline has been reported to help in maintaining cellular osmotic balance, apart from acting as a direct free radical scavenger. It also helps in protecting the activity of various enzymes that eventually help the plant to grow better under stress conditions. [56] observed a statistically non-significant difference in proline content in *Arabidopsis* transgenic line as compared to wild types under control condition, however under stress conditions they reported a significant difference in the proline content of transgenic and wild type plants. These results indicates that *Ecmdar* overexpression in transgenic *Arabidopsis* plants improved redox homeostasis.

**Fig 14:**
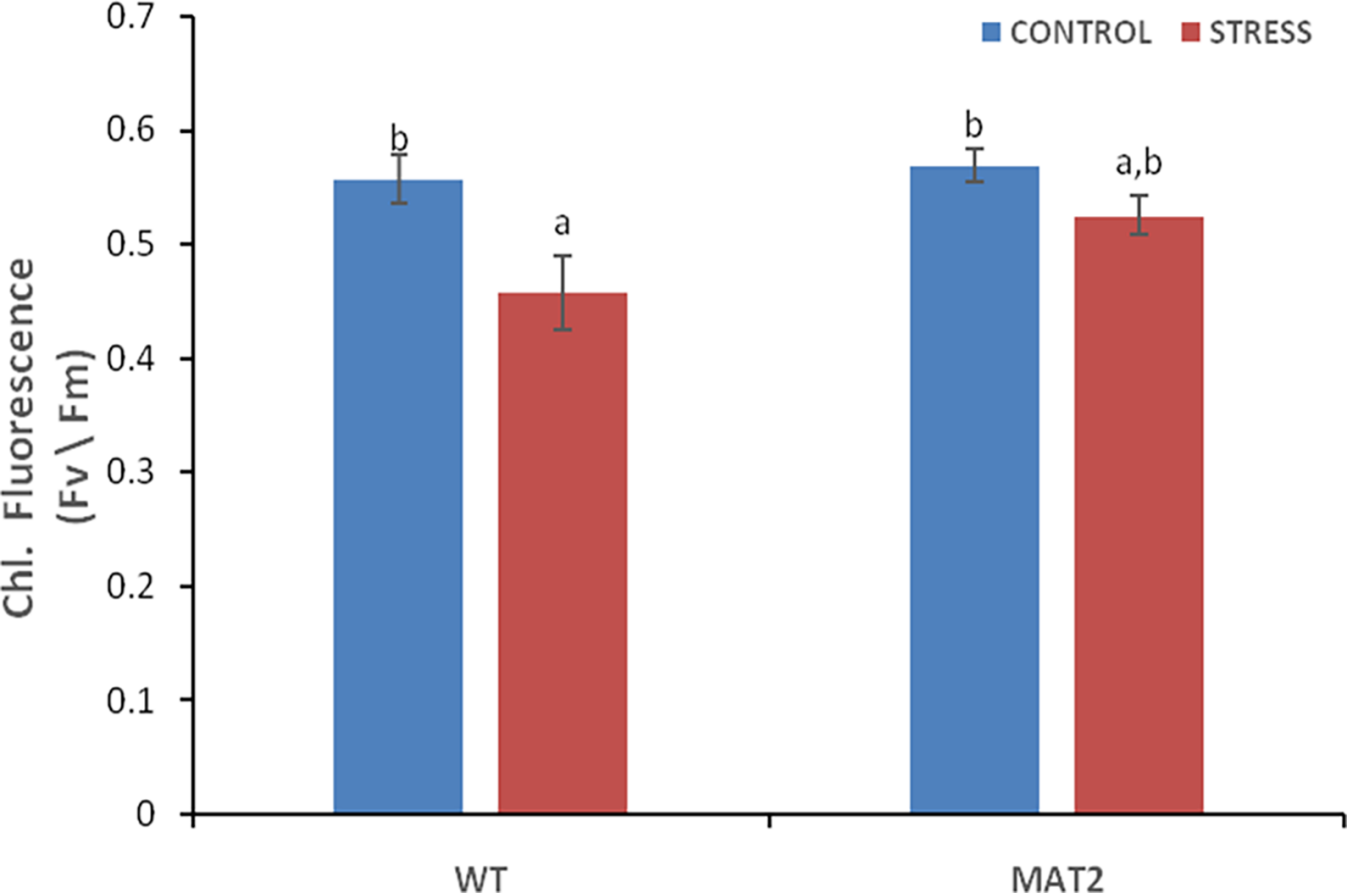
Chlorophyll fluorescence in wild type (WT) and transgenic (MAT2) *Arabidopsis thaliana* line under 100mM NaCl stress. Vertical bars represent Mean±SE. Different letters indicate means that differ significantly (P < 0.05).

Photosynthetic efficiency: The Chlorophyll fluorescence measurements indicate the efficiency of the photo-chemical reactions in dark adapted leaves. The maximal quantum yield of the primary photochemical reactions was adversely affected by salinity stress, in wild type as well as in transgenic lines. However the per cent reduction in *mdar* overexpressing transgenic line (7.7%) was significantly lower than the wild type plants (17.9%). This suggests that electron transport from PSII to PSI in transgenic lines was less affected by stress, as compared to the wild type plants. It has been reported that the amount of chlorophyll fluorescence indicates thylakoid membrane integrity and the relative efficiency of electron transport from PSII to PSI [57].The results signify that the transgenic lines have better stress regulation, and their photosynthetic machinery is less affected by salinity stress and hence are expected to perform better under stress, as compared to the wild type plants (Fig 14).

**Fig 15:**
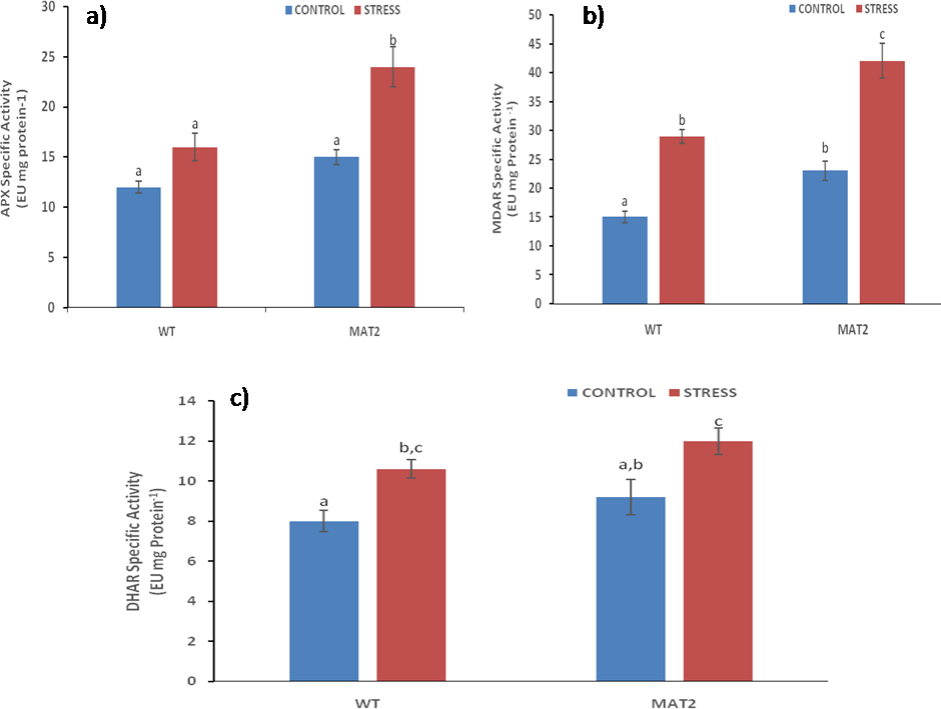
Specific activities of (a) ascorbate peroxidase, (b) monodehydroascorbate reductase and (c) dehydroascorbate reductase in wild type (WT) and transgenic (MAT2) *Arabidopsis thaliana* lines under 0mM (control) and 100mM NaCl. Vertical bars represent Mean±SE. Different letters indicate means that differ significantly (P < 0.05).

Antioxidant enzyme activity: ROS homeostasis is an important component of the antioxidant machinery for protecting cellular metabolism. Plants regulate ROS levels by modulating the activity of ROS scavenging enzymes, such as SOD, APX and MDAR. Moreover, over-expression of specific antioxidant genes has been reported to improve the oxidative stress tolerance profile of transgenic plants. However, the important question is to find out the key regulatory enzyme that can provide meaningful stress tolerance by coordinating the activities of other antioxidant enzymes, through molecular cross talk. In this study, over-expression of *Ecmdar* lead to increased specific activity of ascorbate peroxidase and lowered the specific activity of dehydroascorbate reductase, under salinity stress. Monodehydroascorbate reductase is the enzyme responsible for maintaining the activity of ascorbate peroxidase, by ensuring a continuous supply of reduced ascorbate. As ascorbate peroxidase forms the first line of defense against H_2_O_2_ detoxification [58], sustained supply of reduced ascorbate strengthens the stress response of the plant. A 35% higher APX activity in the transgenic lines as compared to the wild type plants under stress (Fig 15), strengthens this hypothesis.

Further, if not promptly reduced by monodehydroascorbate reductase, the monodehydroascorbate radical can spontaneously dismutate to dehydroascorbate. Reduction of dehydroascorbate to ascorbate requires more energy as compared to the conversion of monodehydroascorbate radical to ascorbate. Therefore, for conserving crucial cellular resources under stress, higher activity of monodehydroascorbate reductase could compensate for dehydroascorbate reductase activity; as recorded in the current experiments wherein transgenic lines recorded only a13% increase in DHAR activity under stress as compared to 82% increase in MDAR activity under stress. Thus overexpression of MDAR can successfully coordinate the activities of the key ascorbate-glutathione pathway enzymes and qualifies to be the candidate gene whose overexpression can provide meaningful stress tolerance potential to the transgenic plants, as observed in the current experiments.

It is clear that monodehydroascorbate reductase enzyme coordinates with ascorbate peroxidase in detoxification of H_2_O_2_, thereby lowering the accumulation of ROS. These ROS, if allowed to accumulate, can potentially damage thylakoid membranes and oxidize the photosynthetic reaction centers. Thus, over-expression of *Ecmdar* successfully lowered the ROS accumulation and reduced the damage to photosynthetic reaction centers, as evident from the improved chlorophyll fluorescence under stress.

## Conclusions

The results obtained in the current investigations conclusively prove that *mdar* is an early responsive gene that responds to various external stimuli like water deficit, salinity and UV radiation stress. The cloned *Ecmdar* gene comprises of a 1437bp coding sequence that encodes a 478 amino acid polypeptide. The EcMDAR protein had 99–85% (nucleotides) and 93–79% (amino acid) similarity with other *mdar* gene and protein sequences. The cloned gene product showed the conserved NAD and FAD binding domains, which contained conserved Cys_69_, Phe_352_, Tyr_173_, Tyr_348_ and His_317_ residues that were found to be essential for its biological activity. Tyr_348_ is the principal amino acid which catalysis the transfer of electron from NAD to MDA. The cloned EcMDAR was found to be a membrane bound peroxisomal protein, containing two transmembrane helices and a C-terminal arginine rich domain, containing the consensus sequence WYGRKRRRW. *Arabidopsis thaliana* transgenic lines overexpressing *Ecmdar*, recorded improved cellular homeostasis under NaCl induced salinity stress, resulting in optimized photosynthetic performance and improved growth. A higher stress induced ascorbate peroxidase activity in transgenic lines, induced by the MDAR overexpression indicates the existence of a cross-talk mechanism between the cellular antioxidant machinery. However, further studies are required for elucidating the secondary messengers involved in this integrated cross-talk. The study successfully underlines the pivotal role of monodehydroascorbate reductase in regulating the antioxidant machinery under stress.

## Supporting information

S1 Fig Pictographic representation of T-DNA region of pEarly Gateway 103 vector. *Ecmdar* vector construct: RB: Right Border, MAS: Mannopine Synthase promoter and terminator, blpR: BASTA resistant gene, 35S CaMV: Cauliflower Mosaic virus promoter, attR: Attachment site, *Ecmdar- Eleusine coracana* monodehydro ascorbate reductase gene, *mgfp- modified gfp*, 6X-His Tag- Histidine tag, OCS- Octapine synthase terminator.
(TIF)

S2 Fig SDS-PAGE analysis of EcMDAR protein expressed in bacterial system. L1- Vector control (supernatant); L2-positive control (pellet); L3- positive control (supernatant); L4-Auto induction (pellet); L5- Auto induction (supernatant); L6- IPTG 0.3mM(pellet); L7- IPTG 0.3mM (supernatant), L8-IPTG 0.8mM (pellet); L9- IPTG 0.8mM (supernatant).
(TIF)

S3 Fig Screening of transformed plants. a) BASTA Selection b) PCR confirmation of BASTA positive plants: L: 1 Kb plus ladder, RC: Reaction control, Positive control: amplicon amplified from recombinant plasmid, Negative control: (wild type Arabidopsis thaliana genomic DNA), 1–7: putative transgenic plants.
(TIF)

## Abbreviations

MDAR: Monodehydroascorbate reductase protein
Ec: *Eleusine coracana*
CDS: Coding DNA Sequence
ROS: reactive oxygen species
