# Supplementary figures and images for: Molecular cloning, in-silico characterization and functional validation of monodehydroascorbate reductase gene in Eleusine coracana
Source: PLoS One. 2017 Nov 27;12(11):e0187793. doi: 10.1371/journal.pone.0187793 (PMC5703496; doi:10.1371/journal.pone.0187793)

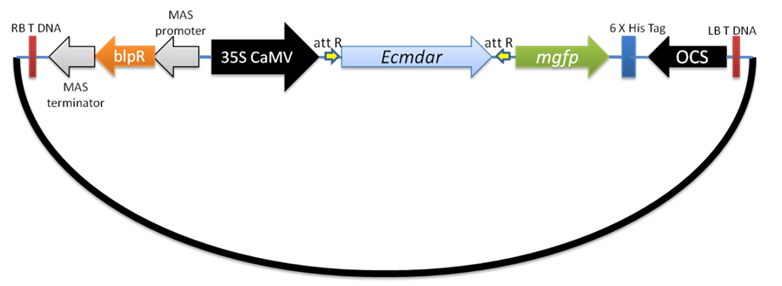

Supplement: S1 Fig — Ecmdar vector construct: RB: Right Border, MAS: Mannopine Synthase promoter and terminator, blpR: BASTA resistant gene, 35S CaMV: Cauliflower Mosaic virus promoter, attR: Attachment site, Ecmdar- Eleusine coracana monodehydro ascorbate reductase gene, mgfp- modified gfp, 6X-His Tag- Histidine tag, OCS- Octapine synthase terminator. (TIF) [file pone.0187793.s001.tif]

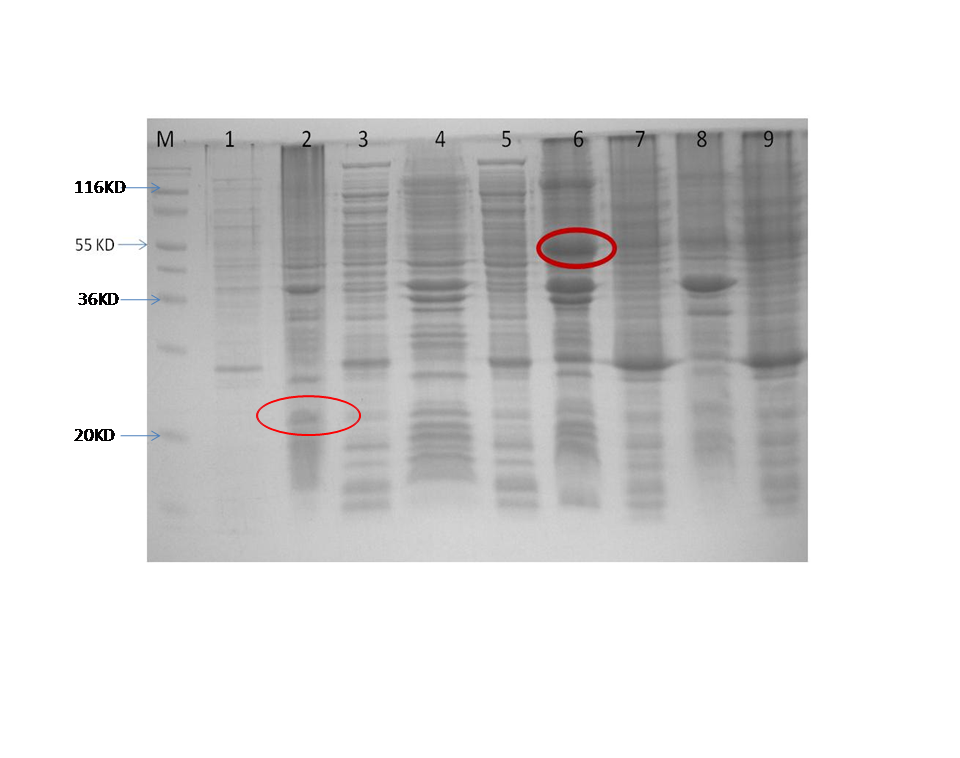

Supplement: S2 Fig — L1- Vector control (supernatant); L2-positive control (pellet); L3- positive control (supernatant); L4-Auto induction (pellet); L5- Auto induction (supernatant); L6- IPTG 0.3mM(pellet); L7- IPTG 0.3mM (supernatant), L8-IPTG 0.8mM (pellet); L9- IPTG 0.8mM (supernatant). (TIF) [file pone.0187793.s002.tif]

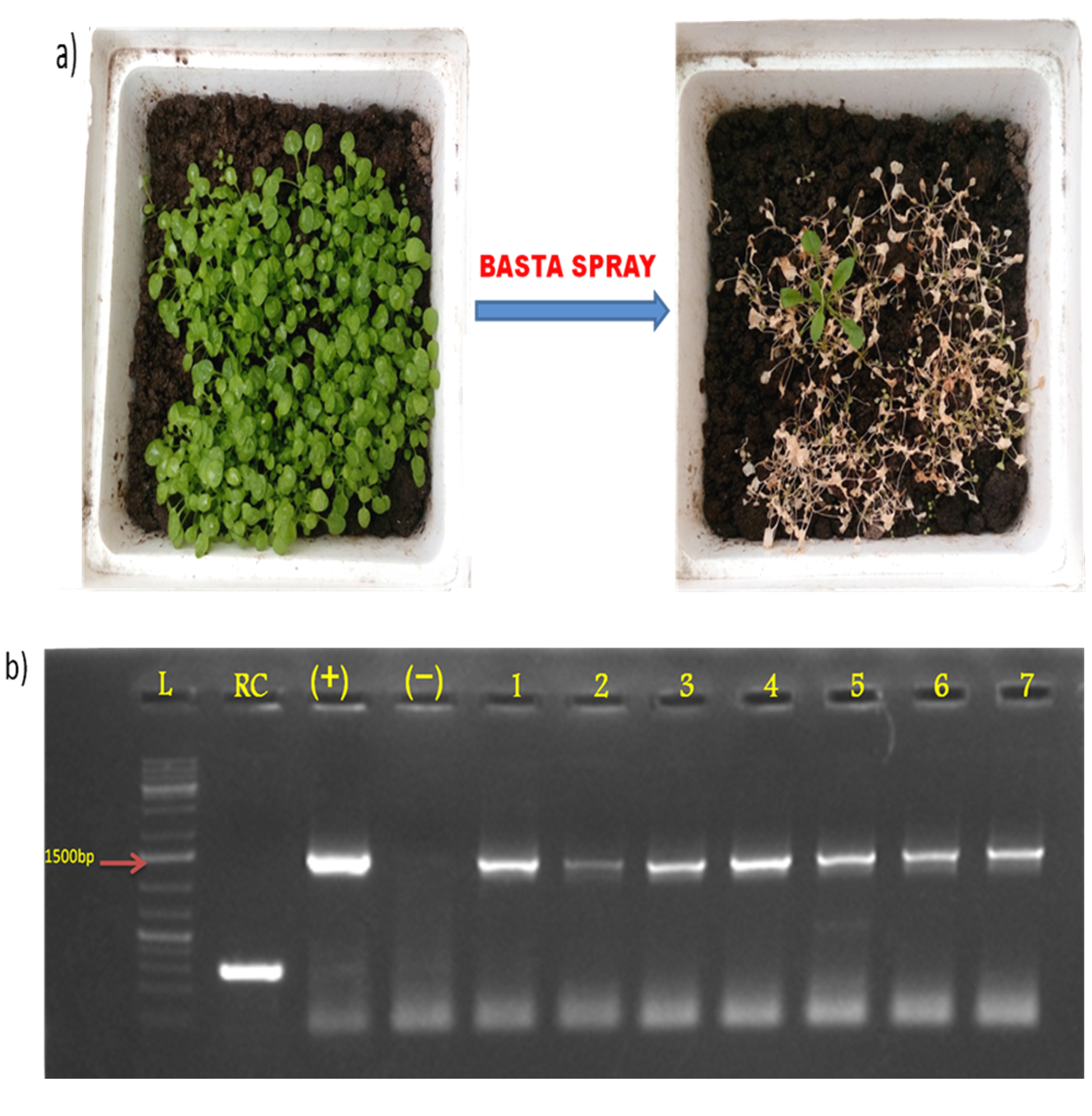

Supplement: S3 Fig — a) BASTA Selection b) PCR confirmation of BASTA positive plants: L: 1 Kb plus ladder, RC: Reaction control, Positive control: amplicon amplified from recombinant plasmid, Negative control: (wild type Arabidopsis thaliana genomic DNA), 1–7: putative transgenic plants. (TIF) [file pone.0187793.s003.tif]
